# [^18^F]Atorvastatin: synthesis of a potential molecular imaging tool for the assessment of statin-related mechanisms of action

**DOI:** 10.1186/s13550-020-00622-4

**Published:** 2020-04-15

**Authors:** Gonçalo S. Clemente, Jens Rickmeier, Inês F. Antunes, Tryfon Zarganes-Tzitzikas, Alexander Dömling, Tobias Ritter, Philip H. Elsinga

**Affiliations:** 1 Department of Nuclear Medicine and Molecular Imaging – University Medical Center Groningen, University of Groningen, Hanzeplein 1, 9713 GZ Groningen, The Netherlands; 2 Max-Planck-Institut für Kohlenforschung, Kaiser-Wilhelm-Platz 1, 45470 Mülheim an der Ruhr, Germany; 3 Department of Drug Design, University of Groningen, Antonius Deusinglaan 1, 9713 AV Groningen, The Netherlands

**Keywords:** Statins, Atorvastatin, HMG-CoA, ^18^F-deoxyfluorination, Fluorine-18, Positron emission tomography

## Abstract

**Background:**

Statins are lipid-lowering agents that inhibit cholesterol synthesis and are clinically used in the primary and secondary prevention of cardiovascular diseases. However, a considerable group of patients does not respond to statin treatment, and the reason for this is still not completely understood. [^18^F]Atorvastatin, the ^18^F-labeled version of one of the most widely prescribed statins, may be a useful tool for statin-related research.

**Results:**

[^18^F]Atorvastatin was synthesized via an optimized ruthenium-mediated late-stage ^18^F-deoxyfluorination. The defluoro-hydroxy precursor was produced via Paal-Knorr pyrrole synthesis and was followed by coordination of the phenol to a ruthenium complex, affording the labeling precursor in approximately 10% overall yield. Optimization and automation of the labeling procedure reliably yielded an injectable solution of [^18^F]atorvastatin in 19% ± 6% (d.c.) with a molar activity of 65 ± 32 GBq·μmol^−1^. Incubation of [^18^F]atorvastatin in human serum did not lead to decomposition. Furthermore, we have shown the ability of [^18^F]atorvastatin to cross the hepatic cell membrane to the cytosolic and microsomal fractions where HMG-CoA reductase is known to be highly expressed. Blocking assays using rat liver sections confirmed the specific binding to HMG-CoA reductase. Autoradiography on rat aorta stimulated to develop atherosclerotic plaques revealed that [^18^F]atorvastatin significantly accumulates in this tissue when compared to the healthy model.

**Conclusions:**

The improved ruthenium-mediated ^18^F-deoxyfluorination procedure overcomes previous hurdles such as the addition of salt additives, the drying steps, or the use of different solvent mixtures at different phases of the process, which increases its practical use, and may allow faster translation to clinical settings. Based on tissue uptake evaluations, [^18^F]atorvastatin showed the potential to be used as a tool for the understanding of the mechanism of action of statins. Further knowledge of the in vivo biodistribution of [^18^F]atorvastatin may help to better understand the origin of off-target effects and potentially allow to distinguish between statin-resistant and non-resistant patients.

## Background

**Fig. 1:**
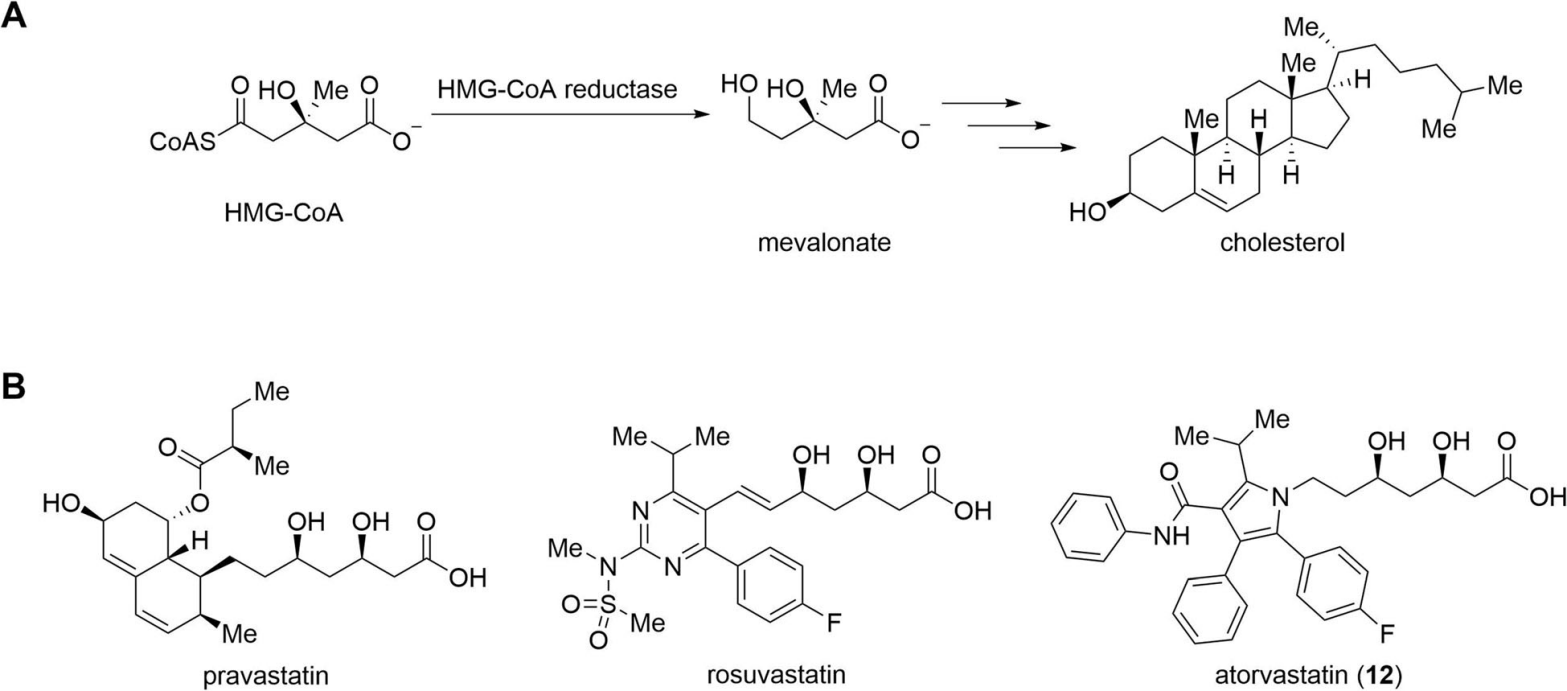
Role of HMG-CoA reductase in the synthesis of cholesterol (**a**) and structure of some of the most clinically used statins (**b**)

Cardiovascular diseases represent one of the leading causes of death globally [1]. Atherosclerosis, a chronic inflammatory pathology characterized by deposition of plaques (a collection of fat, cholesterol, calcium, fibrin, and cellular waste products) in the walls of arteries, is the dominant cause of cardiovascular diseases [2]. The partial rupture and detachment of these plaques increase the risk of clogging the blood flow, which, ultimately, can lead to highly incapacitating or even mortal conditions such as myocardial infarction or cerebrovascular accidents [3]. The exact cause of atherosclerosis remains a subject of discussion and has been connected to several distinct mechanisms, hypotheses, and theories [4–16]. Nonetheless, despite its complexity, atherosclerosis is generally correlated to the levels of cholesterol in plasma [17–22]. HMG-CoA (3-hydroxy-3-methyl-glutaryl-coenzyme A) reductase is involved in the biosynthesis of cholesterol and is subject to feedback regulation by end-products of this same pathway [23–26]. Thus, inhibition of HMG-CoA reductase with statins became an essential strategy for the primary prevention of atherosclerosis. Statins contain a dihydroxycarboxylic acid moiety that mimics and outcompetes the natural substrate molecule HMG-CoA, preventing its reduction to mevalonate and further cholesterol synthesis [27, 28] (Fig. 1a).

In addition to the cholesterol-lowering action, the success of statins became increasingly connected with broader pleiotropic effects [29–32]. Statins are increasingly being associated with potential protective effects on pathologies beyond cardiovascular diseases (e.g., respiratory [33–37], carcinogenic [38–43], viral [44–48], neurodegenerative [49–53]). The exact off-target mechanisms of statins, along with the reason why many patients show resistance to this class of drugs, are still unknown. Therefore, an increase of specific knowledge of the subcellular mechanisms affected by statins and the development of a more sensitive tool to investigate this subject are currently challenging hot topics in medicinal chemistry [54, 55]. High-resolution molecular imaging modalities, such as positron emission tomography (PET), together with sensitive nuclear analytical techniques relying on radiolabeled molecules, can help in a better understanding by mapping the biodistribution profile over time within complex living organisms. To this purpose, we report the ^18^F-labeling of atorvastatin (**12**), one of the most widely used statins in the prevention of cardiovascular risk factors and one of the best-selling drugs in pharmaceutical history [56]. Exchanging the aryl fluoride with its ^18^F radioisotope enables the radiolabeling of atorvastatin without changing its pharmacological properties. However, in the case of atorvastatin, the ^18^F-fluorination of electron-rich arenes is a classical struggle in radiochemistry and has challenges associated with synthesis automation [57]. The fluorine-containing aromatic ring within the atorvastatin structure is electron-rich, and therefore, we use the ruthenium-mediated radiodeoxyfluorination strategy [58–60] to synthesize [^18^F]atorvastatin (**[**^**18**^**F]12**). This radiotracer may have the potential to become an attractive tool for statin-related research, enabling the understanding of cellular and subcellular mechanisms, the identification of off-target activity and, ultimately, allowing to select between statin-resistant and non-resistant patients for targeted therapy.

## Results

### Synthesis of precursors and ^18^F-fluorination strategy

**Scheme 1:**
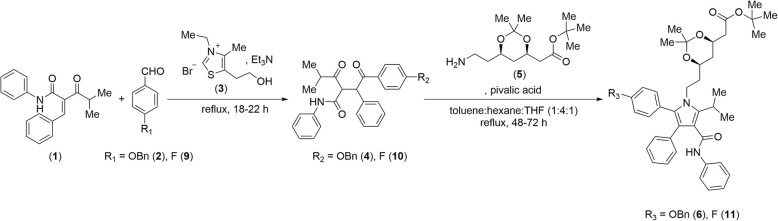
Synthesis of ketal and *tert*-butyl ester side-chain protected benzyl ether pyrrole intermediate **6** and atorvastatin precursor **11**

The synthesis of the benzyl ether pyrrole intermediate **6**, as well as the atorvastatin precursor **11**, was performed by the treatment of the corresponding 1,4-diketone intermediates **4** and **10**, respectively, with the commercially available primary amine **5** in a pivalic acid-catalyzed Paal-Knorr condensation reaction (Scheme 1). The 1,4-diketone intermediates **4** and **10** were synthesized via a Stetter reaction [61, 62]. In summary, the *α,β*-unsaturated ketone **1** was combined with benzaldehydes **2** and **9**, respectively, in the presence of triethylamine and the thiazolium bromide catalyst **3** to afford the corresponding 1,4-diketone intermediates **4** and **10**, respectively. Atorvastatin (**12**) was obtained from its precursor **11** after near quantitative removal of the ketal-ester protecting groups in the side-chain with sequential hydrochloric acid and sodium hydroxide treatment.

**Scheme 2:**
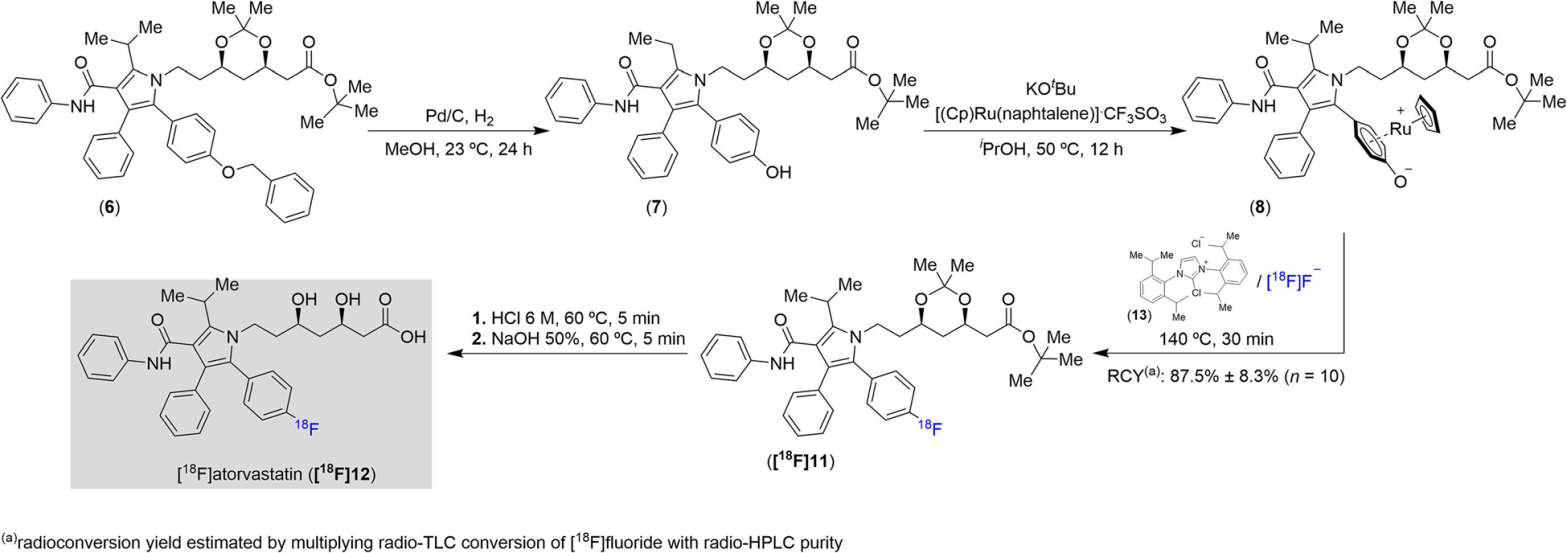
[^18^F]Atorvastatin (**[**^**18**^**F]12**) synthesis approach used in this work

For the radiofluorination of atorvastatin, the benzyl ether pyrrole intermediate **6** was synthesized and subsequently converted to the corresponding phenol **7** by palladium on carbon (Pd/C)-catalyzed hydrogenolysis (Scheme 2). Coordination of **7** to ruthenium (II) decreases its π-electron density, providing **8**, and activates the precursor towards radiodeoxyfluorination [58–60].

### Radiochemistry optimization: improvement and simplification of the Ru-mediated ^18^F-deoxyfluorination

Synthesis of [^18^F]atorvastatin (**[**^**18**^**F]12**) started with conventional trapping of the aqueous [^18^F]fluoride, produced by a cyclotron using the ^18^O(*p*,*n*)^18^F nuclear reaction, on an anion exchange cartridge (Chromafix 45-PS-HCO_3_^−^). The [^18^F]fluoride was then eluted from the cartridge with a solution of the labeling precursor **8** (2.5 μmol), *N*,*N*-bis(2,6-diisopropylphenyl-1-chloroimidazolium chloride (**13**, 6.0 equiv.), and bis(trimethylneopentylammonium) oxalate in a mixture of ethanol:pivalonitrile:veratrole (400 μL, 1:4:4 v:v:v) directly into a reaction vial [60]. The reaction mixture was heated under vigorous stirring at 140 °C for 30 min to afford the key intermediate **[**^**18**^**F]11**. A drawback of this literature procedure is the reduced elution efficiency from the anion exchange cartridge. Besides, there is no commercial supplier of the oxalate salt. This may hinder good manufacturing/radiopharmaceutical/clinical practice (GMP/GRPP/GCP) applications with the synthesized radiotracer as additional analytical evaluations, toxicity tests, and reliable and established production of the additive may be needed to comply with potential clinical trials in human patients [63]. As mentioned in a previous report [58], salt additives tend to reduce the ^18^F-deoxyfluorination yield even though the elution efficiency is increased. Thus, in this work, several readily available additives were evaluated in order to achieve a better elution alternative that can increase the elution efficiency while minimally affecting the ^18^F-deoxyfluorination yield (Table 1).

**Table 1:** Influence of eluent additives in the synthesis of the intermediate product **[**^**18**^**F]11**

Eluent additive	Elution efficiency^a^	TLC conversion	HPLC purity	[^**18**^F]11 yield^b^
Bis(trimethylneopentylammonium) oxalate	75%	83%	80%	50%
Kryptofix 222, K_2_C_2_O_4_	86%	59%	48%	24%
Tetraethylammonium bicarbonate	54%	34%	50%	9%
Tetrabutylammonium chloride	62%	91%	82%	46%
Sodium acetate	12%	10%	15%	0.2%
Silver acetate	67%	29%	82%	16%
Silver triflate	65%	69%	36%	16%
Sodium oxalate	54%	52%	71%	20%
None	42%	85%	90%	32%

^a^Calculated by the ratio between the [^18^F]fluoride trapped in the anion exchange cartridge (45-PS-HCO_3_^−^) and the radioactivity recovered (without reversing the cartridge) in the reaction vial

^b^Non-isolated ^18^F-deoxyfluorination yield based on radio-TLC and radio-HPLC analysis of the crude product and having in consideration the elution efficiency resulting from the salt additive used in relation to the starting radioactivity. The final percentage of **[**^**18**^**F]11** yield was determined by multiplying the elution efficiency with radio-TLC conversion of the starting [^18^F]fluoride and with radio-HPLC purity (*n* ≥ 2)

As expected, most of the tested additives have a negative effect on the ^18^F-deoxyfluorination yield. The use of bis(trimethylneopentylammonium) oxalate [58–60] gave the best overall yields of **[**^**18**^**F]11**. This addition still decreases the ^18^F-deoxyfluorination conversion by approximately 10% when compared to the experiments without an eluent additive. An acceptable alternative to the oxalate seems to be the use of the commercially available tetrabutylammonium chloride, which, despite lowering the elution efficiency, led to a similar **[**^**18**^**F]11** yield.

**Fig. 2:**
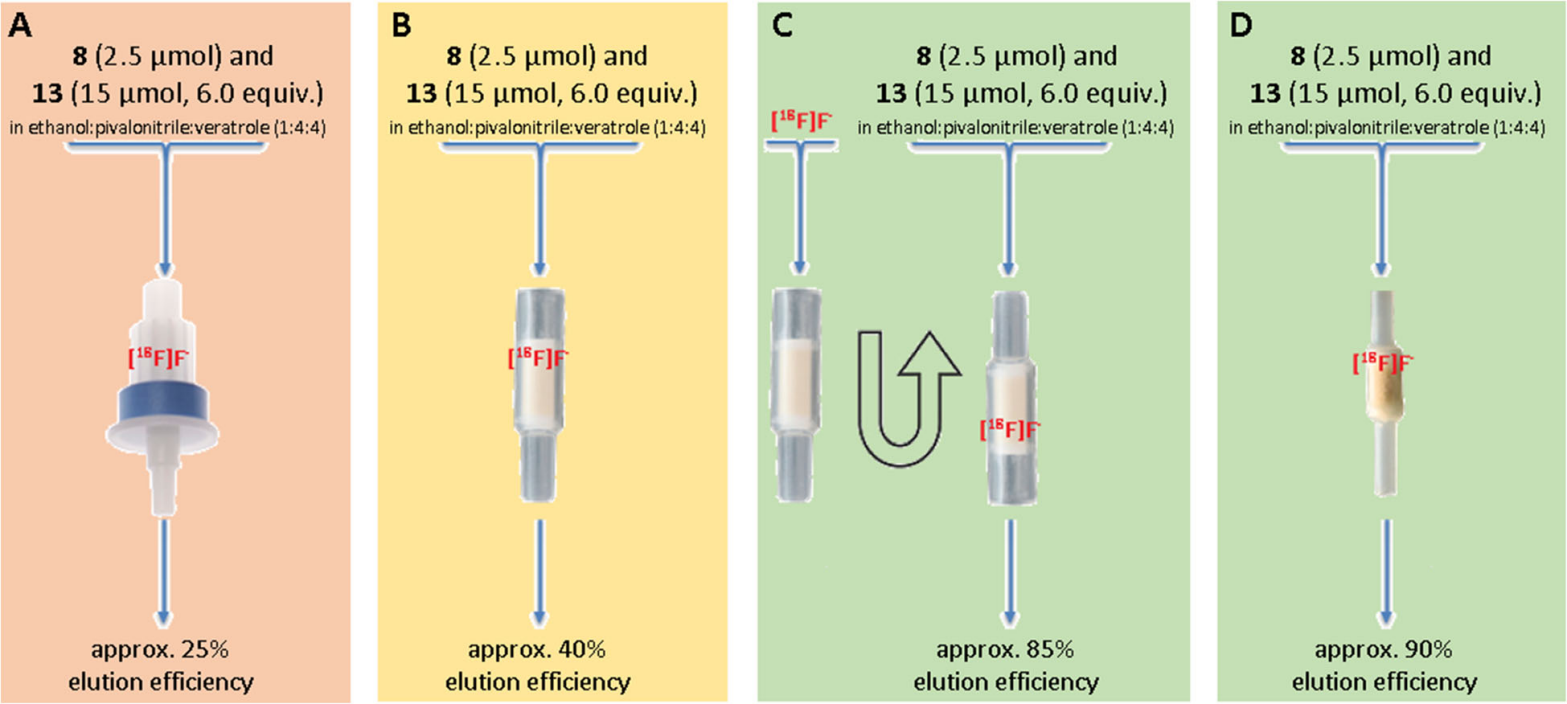
Anion exchange cartridge alternatives tested in this work. **a** Sep-Pak Accell Plus QMA Plus Light Cartridge (Waters). **b** 45-PS-HCO_3_^−^ (Chromafix). **c** Reversed 45-PS-HCO_3_^−^ (Chromafix). **d** Handmade 1/16″ PTFE tubing with MP-1 resin (Biorad)

Further experiments showed that in the absence of an eluent additive, the elution efficiency can be more than doubled by reversely loading and eluting the 45-PS-HCO_3_^−^ cartridge or by replacing this cartridge to a short 1/16″ PTFE tubing filled with approximately 10 mg of a Biorad MP-1 resin (Fig. 2). However, reversing the cartridge cannot easily be implemented in most automated modules. Also, since the MP-1 mini-cartridge is not commercially available and should, therefore, be manually prepared, it might result in significant elution differences from batch to batch, which will affect the activity yield. This led us to the evaluation of different solvent systems (without the use of eluent additives) to increase the elution efficiency (Table 2).

**Table 2:** Influence of the solvent system in the synthesis (without eluent additives) of the intermediate product **[**^**18**^**F]11**

Solvent (400 μL)	Elution efficiency^c^	TLC conversion	HPLC purity	[^**18**^F]11 yield^d^
Ethanol:pivalonitrile:veratrole (1:4:4)	42%	85%	90%	32%
Ethanol:acetonitrile:DMSO (1:4:4)	69%	75%	80%	41%
Veratrole	30%	85%	61%	16%
Pivalonitrile	35%	80%	63%	18%
Dimethyl sulfoxide (DMSO)	57%	68%	64%	25%
Methanol:DMSO (1:2)	86%	64%	72%	40%
Methanol:DMSO (1:3)	86%	95%	92%	75%
Methanol:DMSO (1:3.5)	85%	86%	87%	64%
Methanol:DMSO (1:7)	65%	55%	90%	32%
Butanol:DMSO (1:2)	50%	20%	23%	2%
Butanol:DMSO (1:3)	54%	80%	79%	34%
Ethanol:DMSO (1:3.5)	70%	78%	78%	43%
Water:DMSO (1:17)	36%	85%	83%	25%
Aqueous [^18^F]fluoride:DMSO (3:97)^a^	100% (no cartridge)	92%	91%	84%
Methanol:veratrole (1:3)	90%	94%	97%	82%
Methanol + veratrole:pivalonitrile (1:1)^b^	93% (56%)	85%	94%	74% (45%)

^a^30 μL of aqueous [^18^F]fluoride was directly added (no elution cartridge needed) to the DMSO solution containing **8** and **13** and left to react

^b^300 μL of methanol was used to dissolve **8** and **13** to elute the cartridge. Methanol was then evaporated, and the solvent exchanged to 400 μL of veratrole:pivalonitrile (1:1 v:v) for the reaction. This method, despite having high elution efficiency (93%), showed significant losses (up to 45%) of the eluted [^18^F]fluoride during the evaporation of methanol. Thus, the real efficiency and [^**18**^**F]11** yield is shown in brackets

^c^Calculated by the ratio between the [^18^F]fluoride trapped in the anion exchange cartridge (45-PS-HCO_3_^−^) and the radioactivity recovered (without reversing the cartridge) in the reaction vial

^d^Non-isolated ^18^F-deoxyfluorination yield based on radio-TLC and radio-HPLC analysis of the crude product and having in consideration the elution efficiency resulting from the solvent mixture used in relation to the starting radioactivity. The final percentage of **[**^**18**^**F]11** yield was determined by multiplying the elution efficiency with radio-TLC conversion of the starting [^18^F]fluoride and with radio-HPLC purity (*n* ≥ 2)

By changing the solvents used to dissolve the labeling precursor **8** and the chloroimidazolium chloride **13**, while keeping all other reaction conditions unchanged, we were able to improve not only the elution efficiency but also the yield of the ^18^F-deoxyfluorination to achieve the intermediate product **[**^**18**^**F]11**. Using a mixture of methanol:veratrole (1:3 v:v) or methanol:DMSO (1:3 v:v) provided the best results. However, the mixture with veratrole quickly builds up high pressure in the reaction vial at 140 °C (with some associated radioactivity escape). Thus, replacing veratrole by *dimethylsulfoxide* (DMSO) is the safer choice of solvent. This optimized method boosted and simplified the ^18^F-deoxyfluorination technique by avoiding the addition of salt additives, skipping any need for washing the trapped [^18^F]fluoride, circumventing time-consuming azeotropic drying (with the consequent radioactivity losses by natural decay, evaporation, and unspecific adsorption to the reactor surface) and the use of different solvent mixtures for the various steps of the process (elution and ^18^F-fluorination). The amount of Ru-coordinated precursor **8** was also reduced in relation to the previously reported ^18^F-deoxyfluorination works (2.5 μmol vs. 5.0 μmol) [58–60]. During the optimization work, it became evident that some procedure deviations such as air bubbling instead of stirring, absence of mechanical mixing, increased solvent volumes, larger reaction vials, or flat-shaped bottom ones can decrease the final ^18^F-deoxyfluorination yield. In order to enhance the radiolabeling efficiency, these are features that should be considered when choosing the most suitable radiosynthesis module.

Additionally, an exciting finding is the relative tolerance of ^18^F-deoxyfluorination to the presence of water, which allowed direct fluorination using aqueous [^18^F]fluoride from the cyclotron target without the need for anion exchange cartridges. The drawback is the low volume of aqueous [^18^F]fluoride that can be used, which limits the amount of radioactivity in the reaction. Nevertheless, this is potentially a suitable method for small-scale productions aimed, for example, for in vitro evaluations.

### Automated synthesis of [^18^F]atorvastatin (**[**^**18**^**F]12**)

**Fig. 3:**
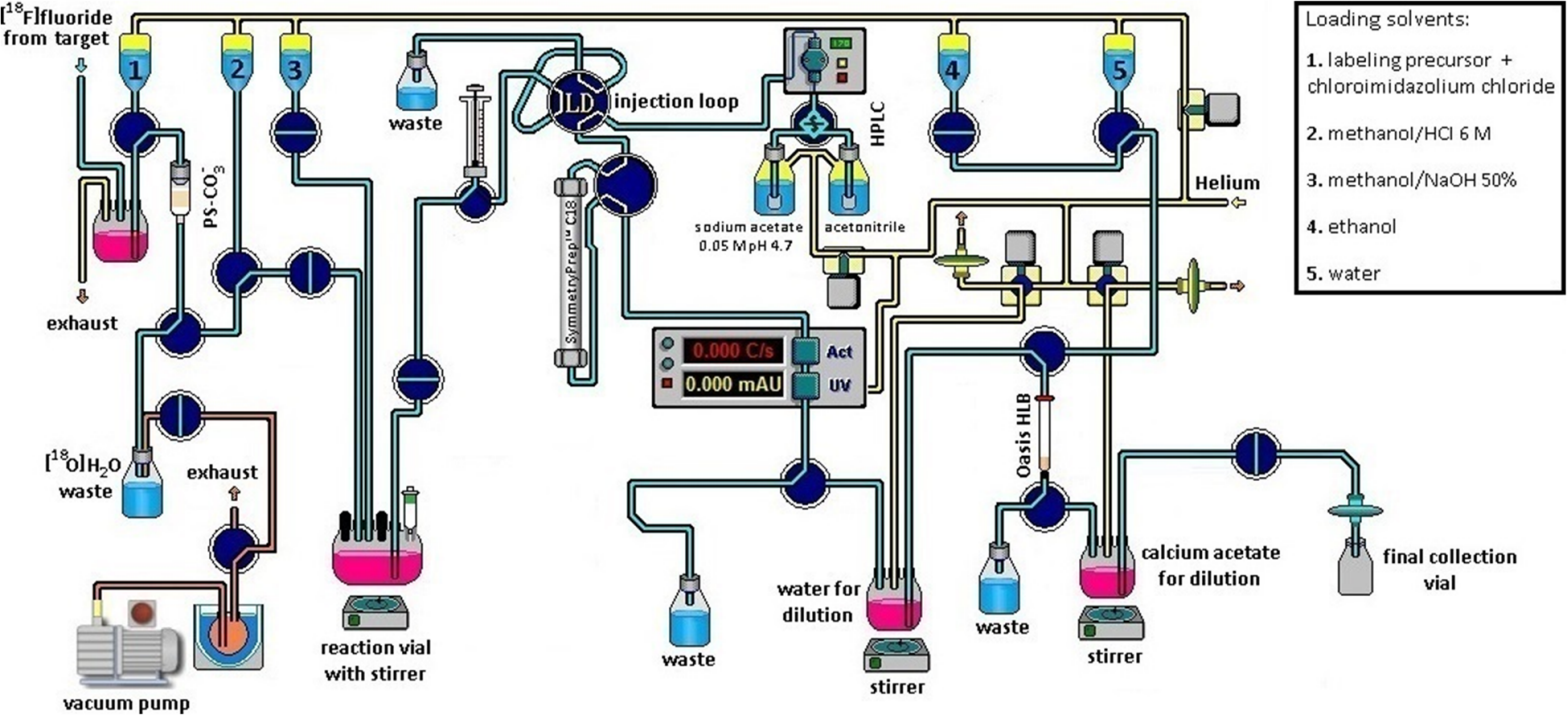
Overall scheme of the full Synthra RNplus setup used

The optimized procedures were translated to the Synthra RNplus radiosynthesizer. Minimal modifications were made to the commercial configuration of the module (Fig. 3). All unused loading ports to the reaction vial were closed with perfluoroalkoxy plugs, except one that was left open with an ethylene tetrafluoroethylene female luer-to-male fitting for connection to an exhaust alumina N cartridge (Waters). In summary, aqueous [^18^F]fluoride was trapped on a Chromafix 45-PS-HCO3^−^ anion exchange cartridge preactivated by sequentially passing 3 mL of K_2_C_2_O_4_ (10 mg/mL) and 2 mL of water and dried with a flow of argon (trapping efficiency, 94% ± 4%; *n* = 10). After drying the resin in the cartridge under helium flow for approximately 1 min, the trapped [^18^F]fluoride was eluted by pushing a solution of the Ru-coordinated labeling precursor **8** (2.5 μmol) and *N*,*N*-bis(2,6-diisopropylphenyl-1-chloroimidazolium chloride (**13**, 6.0 equiv.) in methanol:DMSO (1:3 v:v, 0.4 mL) over 2 min through the cartridge, directly to a reaction vial preheated at 110 °C (elution efficiency, 88 ± 5%; *n* = 10). This preheating seems to avoid the significant radioactivity losses that were seen when rapidly increasing from room temperature to the reaction temperature or when the reaction vial was already set at 140 °C. The reaction was left to stir for 30 min at 140 °C to produce the intermediate **[**^**18**^**F]11** (^18^F-deoxyfluorination yield estimated by multiplying radio-TLC conversion of [^18^F]fluoride with radio-HPLC purity, 87 ± 9%; *n* = 10). The ketal and *tert*-butyl protecting groups were removed by sequentially treating the reaction mixture with 1 mL of methanol:HCl 6 M (49:1 v:v) at 60 °C for 5 min, followed by addition of 0.5 mL of methanol:aqueous NaOH 50% (9:1 v:v) and stirring at 60 °C for another 5 min. HPLC analysis revealed the complete absence of the intermediate **[**^**18**^**F]11** from the reaction mixture. The content of the reaction vial was automatically loaded onto the built-in preparative HPLC (with UV and radioactivity detector) to collect the [^18^F]atorvastatin (**[**^**18**^**F]12**) fraction. Final solid-phase extraction (SPE) enabled the solvent exchange, and **[**^**18**^**F]12** was then reformulated in a physiological and injectable calcium acetate solution to mimic the atorvastatin (**12**) calcium preparation in which the therapeutic dose of this drug is generally administered. The radiochemical yield of [^18^F]atorvastatin (**[**^**18**^**F]12**) was 19 ± 6%, *n* = 10 (d.c.), and the molar activity was 65 ± 32 GBq·μmol^−1^ (*n* = 10) at the end of synthesis. The activity yield achieved for **[**^**18**^**F]12** was 1.3 ± 0.4 GBq (n.d.c.) when starting with 9.6 ± 1.9 GBq of aqueous [^18^F]fluoride produced in cyclotron (*n* = 10).

### Stability tests and characterization of [^18^F]atorvastatin (**[**^**18**^**F]12**)

**Fig. 4:**
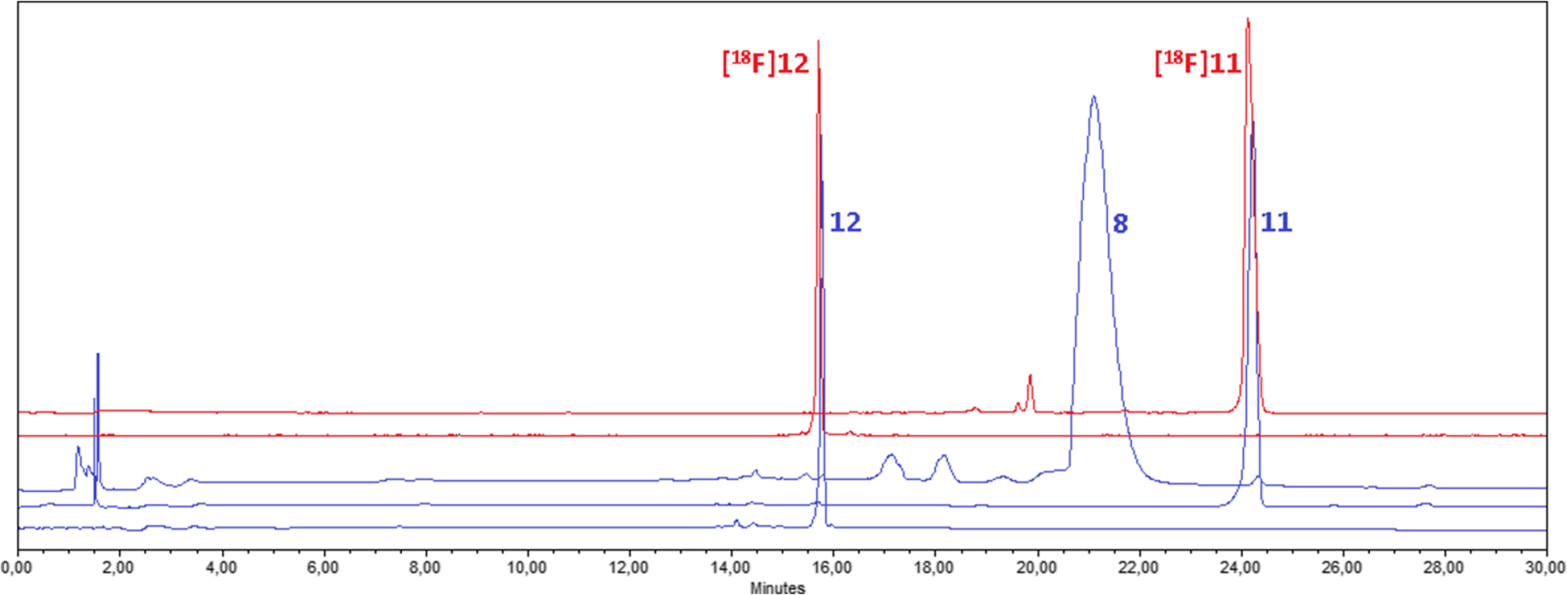
Analytical HPLC profiles (red: γ detector, blue: UV detector) of [^18^F]atorvastatin (**[**^**18**^**F]12**), standard atorvastatin (**12**), radiofluorinated intermediate **[**^**18**^**F]11**, non-radioactive intermediate **11**, and Ru-coordinated labeling precursor **8**

The final purified and formulated radiotracer was compared with the corresponding non-radioactive reference standard by analytical radio-HPLC to confirm identity (Fig. 4). [^18^F]Atorvastatin (**[**^**18**^**F]12**) showed a radiochemical purity always higher than 95%, and no sign of decomposition was observed for at least 4 h in solution at room temperature. Incubation of the radiotracer either in human (collected off the clot from healthy volunteers) or in Wistar rat serum at 37 °C for up to 4 h indicated an in vitro stability of > 95%. Log *D* was measured to confirm the lipophilicity of the radiotracer with the one described for atorvastatin (**12**). An experimental log *D* of 1.61 ± 0.12 (pH 7.4, *n* = 4) was measured, being in accordance with the reported values (log *D* approx. 1.5 [64]).

### Evaluation of [^18^F]atorvastatin (**[**^**18**^**F]12**) in rat liver homogenates and tissue autoradiography

**Fig. 5:**
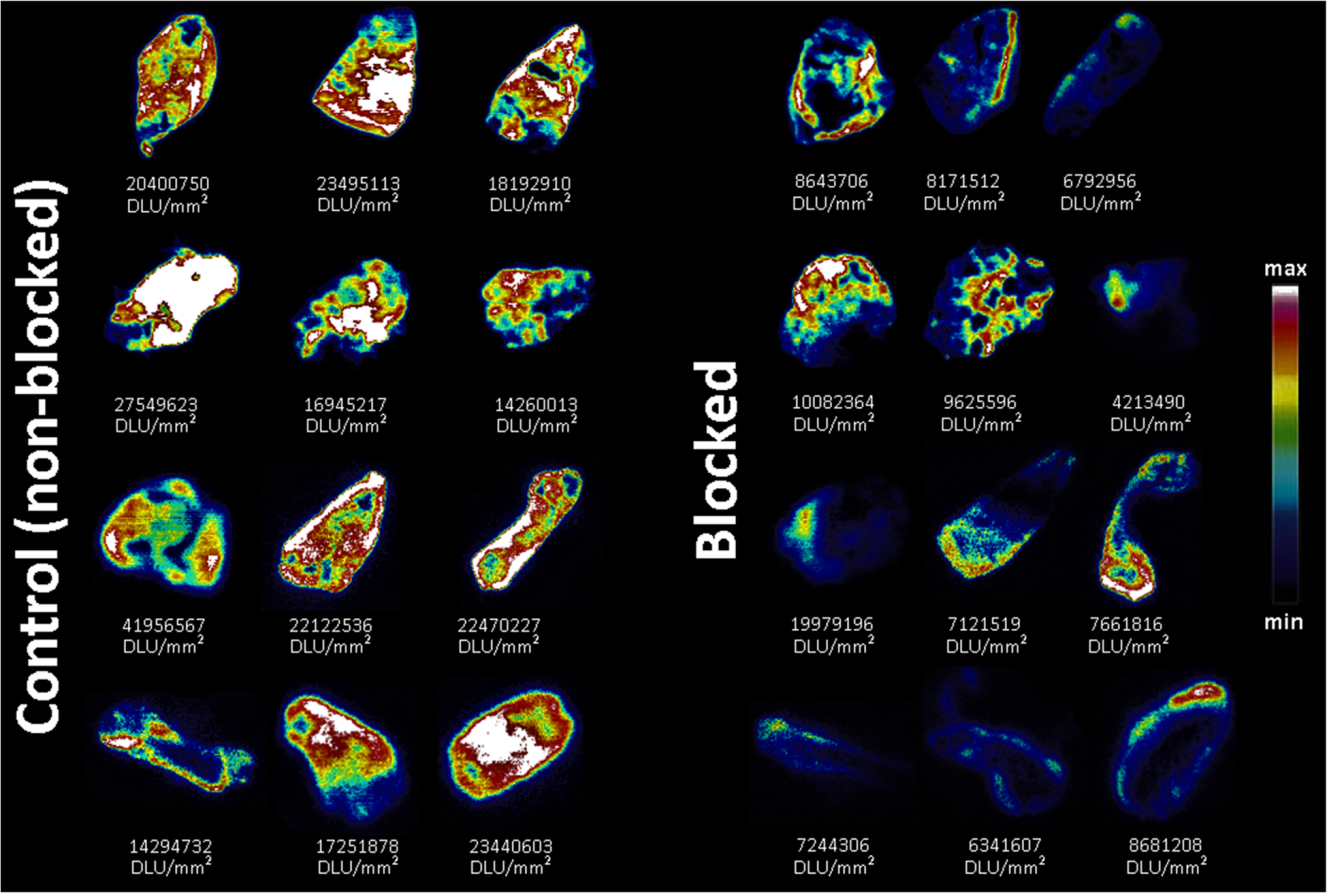
In vitro autoradiography with [^18^F]atorvastatin (**[**^**18**^**F]12**) on rat liver tissue counterparts without (control) and with (blocked) standard atorvastatin (**12**) pre-treatment

HMG-CoA reductase is highly expressed in the liver, where it is subject to hormonal, dietary, and pharmacological regulation [65–67]. Evaluation of [^18^F]atorvastatin (**[**^**18**^**F]12**) in rat liver homogenates revealed that 87% ± 4% (*n* = 4) of the radioactivity is found in the microsomal and cytosolic fractions, which is in accordance with known enzyme distribution [68]. Further radio-TLC assessment showed the absence of degradation and ^18^F-defluorination. Blocking assays using rat liver sections with and without standard atorvastatin (**12**) pre-treatment were performed to evaluate [^18^F]atorvastatin (**[**^**18**^**F]12**) binding selectivity to the HMG-CoA reductase (Fig. 5). The comparison of [^18^F]atorvastatin (**[**^**18**^**F]12**) uptake between non-treated and treated liver sections showed an average 60% ± 8% decrease in the binding of the radiotracer after blocking (*p* < 0.0001).

**Fig. 6:**
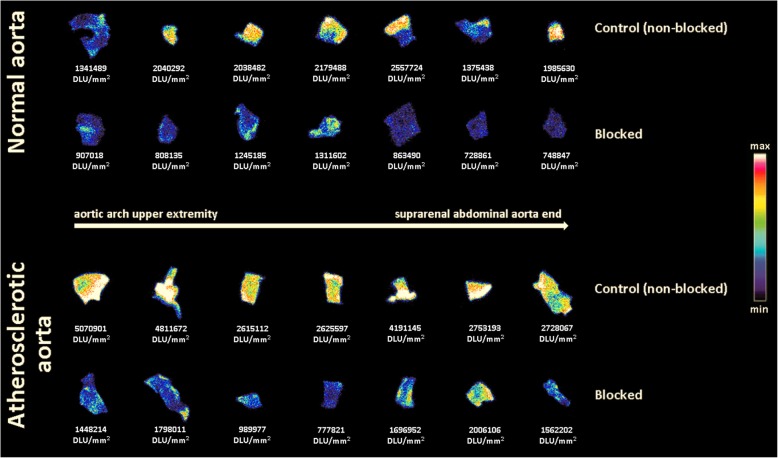
In vitro autoradiography with [^18^F]atorvastatin (**[**^**18**^**F]12**) on the aorta counterparts of a normal and atherosclerotic rat model without (control) and with (blocked) standard atorvastatin (**12**) pre-treatment

The potential of [^18^F]atorvastatin (**[**^**18**^**F]12**) to be used as a PET tracer for atherosclerosis imaging was also preliminarily evaluated by autoradiography of the aorta from a normal and an atherosclerotic rat model. Due to a reduction of lipoprotein clearance, apolipoprotein E-deficient rats fed with high-cholesterol diet tend to develop elevated plasma levels of cholesterol and to form atherosclerotic plaques [69, 70]. Incubation of the aorta excised from a validated rat model for atherosclerosis [71] with the radiotracer showed high [^18^F]atorvastatin (**[**^**18**^**F]12**) uptake in the atherosclerotic aorta (Fig. 6). The radiotracer uptake is reduced when this aorta is pre-treated with standard atorvastatin (**12**) and in the aorta of a normal rat. Average uptake in the atherosclerotic aorta is approximately two times higher than the uptake in the normal aorta (where baseline levels of the HMG-CoA reductase are still present due to its ubiquitous cytoplasmatic expression [72]), being the difference between these groups statistically significant (*p* = 0.004). In the normal aorta, the uptake between control and atorvastatin (**12**) treated groups showed an average 49% ± 13% decrease in the binding efficiency of the radiotracer (*p* = 0.0002). When comparing the uptake results between non-treated and treated atherosclerotic aorta, an average decrease of 56% ± 16% was seen in the binding efficiency of the radiotracer (*p* = 0.0006).

## Discussion

In this work, we explored the late-stage ^18^F-deoxyfluorination of phenols via ruthenium π-complexes, to improve our previous attempt to synthesize the β^+^ emitter version of atorvastatin following the boronic acid pinacol ester cross-coupling late-stage [^18^F]fluorination strategy [73]. This widely clinically prescribed statin has evident benefits in cardiovascular disease prevention and may play a role in other conditions that still require further elucidation. The adjustments used in this work for the Ru-mediated ^18^F-deoxyfluorination strategy proved to be an improvement compared to the previously reported procedures [58–60], including halving the initial labeling precursor amount, avoiding the use of different solvent mixtures and salt additives, and omitting [^18^F]fluoride washing and solvent removal procedures. This, together with the simplicity of the process, the tolerance to a variety of solvents (including aqueous solutions), and the low reaction volumes used, might result in the Ru-mediated deoxyfluorination strategy being suitable for implementation in emerging techniques such as microfluidics-lab-on-a-chip radiochemistry or microdroplet radiosynthesizers. By automating the optimized procedures, this radiofluorination strategy reliably produced [^18^F]atorvastatin (**[**^**18**^**F]12**) in isolated radiochemical yields of 19% ± 6% (d.c.). Within a synthesis time of approximately 80 min, the final injectable physiological solution of the radiotracer was obtained with suitable in vitro stability (at least upon 4 h), radiochemical purity (> 95%), and molar activity (65 ± 32 GBq·μmol^−1^) to evaluate its potential to be used as a PET imaging agent for atherosclerosis.

The access to a radiolabeled analog of atorvastatin allows the mapping and quantification of the radiotracer uptake either in cellular and subcellular compartments or in living complex organisms through high sensitive nuclear analytical and imaging techniques. [^18^F]Atorvastatin-PET might become a relevant research tool for the assessment of statin-related mechanisms of action and to discriminate between responsive and non-responsive patients. Standard atorvastatin (**12**) is known to occupy the binding site of HMG-CoA reductase with high affinity (K_i_ approx. 14 nM [27]) and effectiveness (IC_50_ approx. 8 nM [74]). Thus, the pre-treatment of the liver sections with atorvastatin (**12**) hinders the access of additional substrate to the active site. By incubating rat liver sections, widely known to highly express HMG-CoA reductase [65–67], with [^18^F]atorvastatin (**[**^**18**^**F]12**), the expected selective binding to this enzyme was confirmed since a statistically significant decrease of the uptake was observed when the hepatic tissue was pre-treated with non-radioactive atorvastatin (**12**).

HMG-CoA reductase is known to be overexpressed in atherosclerotic plaques, and its levels are correlated to plaque instability and contribute to the risk of partial rupture and detachment [75]. To evaluate the potential of [^18^F]atorvastatin (**[**^**18**^**F]12**) as a PET tracer for atherosclerosis, an autoradiography imaging study was performed on an excised aorta from an apolipoprotein E-deficient rat model designed to develop atherosclerotic plaques [71]. The uptake of the radiotracer was statistically significant higher in atherosclerotic aorta when compared to a healthy rat aorta. Furthermore, the pre-treatment with the reference compound **12** blocked [^18^F]atorvastatin (**[**^**18**^**F]12**) binding, which supported the potential of this PET tracer to be used for the specific detection of atherosclerotic plaques. As a result of several decades in clinical use, the toxicological profile of atorvastatin (**12**) is widely known. Thus, first in human studies that are already being envisaged at our Nuclear Medicine Department are not being hampered by toxicity issues.

## Conclusions

We have optimized the late-stage Ru-mediated ^18^F-deoxyfluorination strategy performing the synthesis in an automated module (*n* = 10) with suitable radiochemical purity (> 95%) and molar activity (65 ± 32 GBq·μmol^−1^). The affinity of [^18^F]atorvastatin (**[**^**18**^**F]12**) to HMG-CoA reductase and its potential to be used as a PET tracer for atherosclerosis was confirmed in rat liver samples and by comparison of the radiotracer uptake in the aorta of a normal and atherosclerotic rat model. This radiotracer can be a useful tool for the in vitro assessment of statin-related mechanisms of action. Future clinical trials may also be facilitated, as its toxicological profile is already widely characterized due to the extensive clinical use of the non-radioactive analog.

## Methods

### Chemical synthesis

The syntheses of atorvastatin (**12**), labeling precursor **8**, and all intermediate compounds are described and characterized in the Supporting Information file.

### General procedure for the radiosynthesis of atorvastatin (**[**^**18**^**F]12**) by Ru-mediated deoxyfluorination

Cyclotron-produced aqueous [^18^F]fluoride was loaded onto a polystyrene-divinylbenzene copolymers-based HCO_3_^−^ anion exchange cartridge (Chromafix 45-PS-HCO3^−^) preconditioned by sequentially pushing 3 mL of K_2_C_2_O_4_ (10 mg/mL) and 2 mL of water and dried with a flow of Ar. Ru-coordinated labeling precursor **8** (2.5 μmol) and *N*,*N*-bis(2,6-diisopropylphenyl-1-chloroimidazolium chloride (**13**, 6.0 equiv.), with or without a salt additive (see Table 1), were dissolved in 400 μL of a solvent (see Table 2). This solution was used to elute the [^18^F]fluoride directly to a 3-mL glass Wheaton reaction V-vial or a 5-mL glass reactor from a Synthra RNplus radiosynthesizer (containing a stirring bar). The reaction mixture was left under vigorous stirring at 140 °C for 30 min. The intermediate species **[**^**18**^**F]11** was analyzed by radio-TLC (TLC-SG developed with hexane:ethyl acetate (1:1 v:v), Rf ([^18^F]F^-^) = 0.0–0.1 and Rf ([^18^F]11) = 0.8–0–9) and radio-HPLC (SymmetryPrep^TM^ C18 7 μm 7.8 × 300 mm; A, sodium acetate 0.05 M pH 4.7; B, acetonitrile; 0–4 min, 90% A; 4–15 min, 90% A to 20% A; 15–25 min, 20% A to 5% A; 25–33 min, 5% A; 33–34 min, 5% A to 90% A; 34–35 min, 90% A; flow, 6 mL/min; Rt ([^18^F]11) ≈ 24 min). To produce the final product **[**^**18**^**F]12**, 1 mL of a methanol:HCl 6 M (49:1 v:v) solution was added to the reaction mixture and left to stir at 60 °C for 5 min. Subsequently, 0.5 mL of a methanol:aqueous NaOH 50% (9:1 v:v) solution was added to the reaction mixture and left to stir at 60 °C for 5 min. Production of **[**^**18**^**F]12** was assessed by radio-TLC (TLC-SG developed with ethanol:sodium phosphate 0.1 M pH 7.4 (65:35 v:v), Rf ([^18^F]F^-^) = 0.0–0.1, Rf ([^18^F]11) = 0.5–0.6, and Rf ([^18^F]12) = 0.8–0.9) and radio-HPLC (Rt ([^18^F]12) ≈ 16 min). Final reformulated product was obtained after collecting the **[**^**18**^**F]12** fraction by radio-HPLC (SymmetryPrep^TM^ C18 7 μm 7.8 × 300 mm; A, sodium acetate 0.05 M pH 4.7; B, acetonitrile; 0–4 min, 90% A; 4–15 min, 90% A to 20% A; 15–25 min, 20% A to 5% A; 25–33 min, 5% A; 33–34 min, 5% A to 90% A; 34–35 min, 90% A; flow, 6 mL/min; Rt ([^18^F]12) ≈ 16 min; Rt ([^18^F]11) ≈ 24 min) and diluting it in 45 mL of water. This bulk solution was then passed through an Oasis HLB 1 cc cartridge (30 mg sorbent, Waters) to efficiently trap **[**^**18**^**F]12**. The final radiotracer was then washed with 10 mL of water and recovered with 0.5 mL of ethanol. Final dilution in 5 mL of calcium acetate 0.05 M pH 7.0 resulted in an isotonic and injectable [^18^F]atorvastatin (**[**^**18**^**F]12**) solution. For the quality control (QC) of the final radiotracer and assessment of the molar activity, a radio-UPLC system was used (ACQUITY HSS T3 1.8 μm 3.0 × 50 mm column; A, sodium acetate 0.01 M in H_2_O:MeOH:ACN 9:0.6:0.4 v:v:v; B, sodium acetate 0.01 M in H_2_O:MeOH:ACN 1:5.4:3.6 v:v:v; 0–2 min, 100% A; 2–5 min, 100% A to 40% A; 5–6 min, 40% to 0% A; 6–9 min, 0% A; 9–10 min, 0 to 100% A; flow, 0.8 mL/min; UV 244 nm; Rt ([^18^F]12) ≈ 6.2 min).

### Partition coefficient and stability evaluation

The log *D* was measured to determine the lipophilicity of [^18^F]atorvastatin (**[**^**18**^**F]12**). The final reformulated radiotracer (100 μL, in about 10% ethanol) was dissolved in a mixture of 410 μL PBS (pH 7.4) and 490 μL *n*-octanol in a microcentrifuge tube. This mixture was strongly vortexed at room temperature and then centrifuged at 3000 rpm for 5 min. Triplicate samples from both organic and aqueous phases were collected and measured on a ɣ-counter. The log *D* value was reported as the average ratio between the number of counts in the *n*-octanol (upper layer) and PBS (lower layer) obtained in 4 independent measurements.

For the in vitro stability tests, final reformulated **[**^**18**^**F]12** was left at room temperature and analyzed by radio-HPLC and radio-TLC at distinct time points up to 4 h. The in vitro stability assays were performed by incubating 30 μL of **[**^**18**^**F]12** (approx. 1.5 MBq) in 0.3 mL of both human and Wistar rat serum, at 37 °C, up to 4 h and analyzed by radio-TLC and radio-HPLC (in the latter case after protein precipitation with acetonitrile) at various time points. All experiments were done in triplicate.

### Liver homogenate assay

Livers from Wistar rats were harvested, immediately frozen in liquid nitrogen, and conserved at − 80 °C. For the binding assay, tris-HCl (0.05 M, pH 7.4) was added to the thawed rat livers to obtain a final concentration of 20 mg/mL. The mixture was homogenized, using a Heidolph DIAX 600 homogenizer, while being cooled in an ice bath. Liver homogenate batches were stored at − 80 °C. For the binding assay, 300 μL of rat liver homogenate or Tris-HCl solution (for evaluation of the non-specific binding (NSB) fraction of the radiotracer to the tube walls) was added to each centrifuge tube. Tris-HCl solution containing 0.3% human serum albumin was added to reach a final volume of 475 μL and left for pre-incubation for 15 min at room temperature. Then, 25 μL of [^18^F]atorvastatin (**[**^**18**^**F]12**, approx. 1 MBq) was added to each assay tube, briefly vortexed, and then incubated for 60 min under gentle shaking at room temperature. Incubation was terminated by centrifuging (Rotanta 46RS Hettich zentrifugen) the assay tubes at 4 °C for 15 min (7500 rpm). The supernatant (S9 fraction containing cytosol and microsomes) of each tube was transferred to new tubes leaving the pellet (cell debris) in the original tube. The content of NSB assay tubes was also pipetted out to new tubes. All assay tubes were measured on a Wallac Wizard 1480 ɣ-counter to calculate the percentage of radioactivity in each phase (S9 or pellet) with NSB percentage being subtracted to the pellet results.

### Autoradiographic imaging of rat liver

Harvested livers from four Wistar rats (*n* = 4) were sectioned in three thin (1–2 mm) sagittal slices (1–2 mm). To reduce the intervariability, each liver slice was divided into two counterparts and distributed equally between the control and blocking groups (*n* = 4). The sections of each group were impregnated in 3.3 mL of phosphate-buffered saline (PBS) enriched with glucose (5.6 mM), MgCl_2_ (0.49 mM), and CaCl_2_ (0.68 mM) previously warmed at 37 °C. To the control group, 100 μL of DMSO:polyethylene glycol sorbitan monolaurate (9:1 v:v) was added. To the blocking group, 100 μL of atorvastatin (2 mg in DMSO:polyethylene glycol sorbitan monolaurate 9:1 v:v) was added to reach a total concentration of 1 mM with a final volume of 3.5 mL. All liver sections were left to incubate for 30 min. After the incubation time, 100 μL of [^18^F]atorvastatin (**[**^**18**^**F]12**, approx. 4 MBq) was added to each group and left to incubate for 1 h. Finally, the total volume of liquid in all groups was carefully pipetted out and the liver slices were washed 3 times with 3 mL of cold PBS followed by 3 mL of iced water. The slices were then carefully dried and imaged with a GE Healthcare Amersham Typhoon autoradiography system. The acquired data were analyzed with the OptiQuant 03.00 software to quantify the radiotracer uptake in digital luminescence units (DLU).

### Autoradiographic imaging of rat normal and atherosclerotic aorta

Harvested aortas from a healthy Wistar rat and from an apolipoprotein E-deficient Wistar rat model for atherosclerosis [71] were transversely sectioned in 7 segments each from the aortic arch to the suprarenal abdominal aorta end. To reduce sample intervariability, since it is not likely that the distribution of atherosclerotic plaque is homogeneous throughout the total aortic length, each segment was then opened and divided with a sagittal cut in two counterparts. Each counterpart of the same segment was then distributed between two study groups: non-blocked (control) and blocked with atorvastatin. The exposed lumen was finally washed with PBS to guarantee that any potential trace of blood was cleaned out. Then, each aorta section was impregnated in 180 μL of PBS enriched with glucose (5.6 mM), MgCl_2_ (0.49 mM), and CaCl_2_ (0.68 mM) previously warmed at 37 °C. To the control group, 10 μL of DMSO:polyethylene glycol sorbitan monolaurate (9:1 v:v) was added. To the blocking group, 10 μL of atorvastatin (0.1 mg in DMSO:polyethylene glycol sorbitan monolaurate 9:1 v:v) was added to reach a total concentration of 1 mM when in the final 200 μL. All aorta sections were left to incubate for 30 min. After the incubation time, 10 μL of [^18^F]atorvastatin (**[**^**18**^**F]12**, approx. 0.4 MBq) was added to each group and left to incubate for 1 h. Finally, the total volume of liquid was carefully pipetted out and each aorta section was washed 3 times with 250 μL of cold PBS followed by 250 μL of iced water. The slices were then carefully dried and imaged with a GE Healthcare Amersham Typhoon autoradiography system. The acquired data were analyzed with the OptiQuant 03.00 software to quantify the radiotracer uptake in digital luminescence units (DLU).

### Statistics

Data are expressed as the mean ± standard deviation (SD). Unpaired two-tailed *t* tests were used for statistical evaluations. A *p* < 0.01 was considered statistically significant. Statistical analyses of data were performed using GraphPad Prism version 6.01 for Windows (GraphPad Software, La Jolla, CA, USA).

## Supplementary information

**Additional file 1:.** Supporting Information file.

## Data Availability

Samples of the compounds, and datasets used and/or analyzed during the current study are available from the authors on reasonable request. The data that support the findings of this study are included in this published article and its supplementary information files

## Abbreviations

d.c.: Decay corrected
DLU: Digital luminescence units
DMSO: Dimethylsulfoxide
GCP: Good clinical practice
GMP: Good manufacturing practice
GRPP: Good radiopharmacy practice
HMG-CoA: 3-Hydroxy-3-methyl-glutaryl-coenzyme A
HPLC: High-performance liquid chromatography
IC_50_: Half-maximal inhibitory concentration
K_i_: Inhibitory constant
LD_50_: Median lethal dose
LDL: Low-density lipoprotein
MP-1: Macroporous equivalent of analytical grade 1 resin
n.d.c.: Non-decay corrected
NSB: Non-specific binding
PBS: Phosphate-buffered saline (pH 7.4)
PET: Positron emission tomography
PTFE: Polytetrafluoroethylene
QC: Quality control
Rf: Retention factor
Rt: Retention time
SPE: Solid-phase extraction
TLC: Thin-layer chromatography
TLC-SG: Thin-layer chromatography paper impregnated with a silica gel
UPLC: Ultra-performance liquid chromatography
UV: Ultraviolet

## References

[1] Roth GA, Johnson C, Abajobir A, Abd-Allah F, Abera SF, Abyu G (2017). Global, regional, and national burden of cardiovascular diseases for 10 causes, 1990 to 2015. J Am Chem Soc..

[2] Frostegård J (2013). Immunity, atherosclerosis and cardiovascular disease. BMC Med.

[3] Poston RN (2019). Atherosclerosis: integration of its pathogenesis as a self-perpetuating propagating inflammation: a review. Cardiovasc Endocrinol Metab..

[4] Witztum JL (1994). The oxidation hypothesis of atherosclerosis. Lancet (London, England).

[5] Meyers DG (1996). The iron hypothesis—does iron cause atherosclerosis?. Clin Cardiol..

[6] Kuvin JT, Kimmelstiel CD (1999). Infectious causes of atherosclerosis. American Heart Journal..

[7] Stehbens WE (1999). The oxidative stress hypothesis of atherosclerosis: cause or product?. Med Hypotheses..

[8] Lee SA, Amis TC, Byth K, Larcos G, Kairaitis K, Robinson TD (2008). Heavy snoring as a cause of carotid artery atherosclerosis. Sleep..

[9] Verhagen SN, Visseren FLJ (2011). Perivascular adipose tissue as a cause of atherosclerosis. Atherosclerosis..

[10] Sarathi M, Ashley U, Lingyun W, Rui W (2014). Hydrogen sulfide and the pathogenesis of atherosclerosis. Antioxid Redox Signal..

[11] McCully KS (2015). Homocysteine and the pathogenesis of atherosclerosis. Expert Rev Clin Pharmacol..

[12] Mimura J, Itoh K (2015). Role of Nrf2 in the pathogenesis of atherosclerosis. Free Radic Biol Med..

[13] Stancel N, Chen C-C, Ke L-Y, Chu C-S, Lu J, Sawamura T (2015). Interplay between CRP, atherogenic LDL, and LOX-1 and its potential role in the pathogenesis of atherosclerosis. Clin Chem..

[14] Vijayvergiya R, Vadivelu R (2015). Role of Helicobacter pylori infection in pathogenesis of atherosclerosis. World J Cardiol..

[15] Frieri M, Stampfl H (2016). Systemic lupus erythematosus and atherosclerosis: review of the literature. Autoimmun Rev..

[16] Forstermann U, Xia N, Li H (2017). Roles of vascular oxidative stress and nitric oxide in the pathogenesis of atherosclerosis. Circulation research..

[17] Kim YR, Han KH (2013). Familial hypercholesterolemia and the atherosclerotic disease. Korean Circ J..

[18] Berger S, Raman G, Vishwanathan R, Jacques PF, Johnson EJ (2015). Dietary cholesterol and cardiovascular disease: a systematic review and meta-analysis. Am J Clin Nutr..

[19] Chistiakov DA, Bobryshev YV, Orekhov AN (2016). Macrophage-mediated cholesterol handling in atherosclerosis. J Cell Mol Med..

[20] Zárate A, Manuel-Apolinar L, Saucedo R, Hernández-Valencia M, Basurto L (2016). Hypercholesterolemia as a risk factor for cardiovascular disease: current controversial therapeutic management. Arch Med Res..

[21] Peters SAE, Singhateh Y, Mackay D, Huxley RR, Woodward M (2016). Total cholesterol as a risk factor for coronary heart disease and stroke in women compared with men: a systematic review and meta-analysis. Atherosclerosis..

[22] Borén J, Williams KJ (2016). The central role of arterial retention of cholesterol-rich apolipoprotein-B-containing lipoproteins in the pathogenesis of atherosclerosis: a triumph of simplicity. Curr Opin Lipidol..

[23] Honda A, Salen G, Honda M, Batta AK, Tint GS, Xu G (2000). 3-Hydroxy-3-methylglutaryl-coenzyme A reductase activity is inhibited by cholesterol and up-regulated by sitosterol in sitosterolemic fibroblasts. J Lab Clin Med..

[24] Friesen JA, Rodwell VW (2004). The 3-hydroxy-3-methylglutaryl coenzyme-A (HMG-CoA) reductases. Genome Biol..

[25] Buhaescu I, Izzedine H (2007). Mevalonate pathway: a review of clinical and therapeutical implications. Clin Biochem..

[26] DeBose-Boyd RA (2008). Feedback regulation of cholesterol synthesis: sterol-accelerated ubiquitination and degradation of HMG CoA reductase. Cell Res..

[27] Carbonell T, Freire E (2005). Binding thermodynamics of statins to hmg-coa reductase. biochemistry.

[28] Endo A (2010). A historical perspective on the discovery of statins. Proc Jpn Acad Ser B Phys Biol Sci..

[29] Feldstein CA (2010). Statins in hypertension: are they a new class of antihypertensive agents?. American journal of therapeutics..

[30] Antonopoulos AS, Margaritis M, Lee R, Channon K, Antoniades C (2012). Statins as anti-inflammatory agents in atherogenesis: molecular mechanisms and lessons from the recent clinical trials. Curr Pharm Des..

[31] Rizzo M, Montalto G, Banach M (2012). The effects of statins on blood pressure: current knowledge and future perspectives. Arch Med Sci..

[32] Oesterle A, Laufs U, Liao JK (2017). Pleiotropic effects of statins on the cardiovascular system. Circulation research..

[33] Yuan S (2015). Statins may decrease the fatality rate of middle east respiratory syndrome infection. mBio.

[34] Xiong B, Wang C, Tan J, Cao Y, Zou Y, Yao Y (2016). Statins for the prevention and treatment of acute lung injury and acute respiratory distress syndrome: a systematic review and meta-analysis. Respirology..

[35] Thomson NC (2017). Clinical studies of statins in asthma and COPD. Current molecular pharmacology..

[36] So JY, Dhungana S, Beros JJ, Criner GJ (2018). Statins in the treatment of COPD and asthma—where do we stand?. Curr Opin Pharmacol..

[37] Melo AC, Cattani-Cavalieri I, Barroso MV, Quesnot N, Gitirana LB, Lanzetti M (2018). Atorvastatin dose-dependently promotes mouse lung repair after emphysema induced by elastase. Biomed Pharmacother..

[38] Ahern TP, Lash TL, Damkier P, Christiansen PM, Cronin-Fenton DP (2014). Statins and breast cancer prognosis: evidence and opportunities. Lancet Oncol..

[39] Pisanti S, Picardi P, Ciaglia E, D’Alessandro A, Bifulco M (2014). Novel prospects of statins as therapeutic agents in cancer. Pharmacol Res..

[40] Nevadunsky NS, Van Arsdale A, Strickler HD, Spoozak LA, Moadel A, Kaur G (2015). Association between statin use and endometrial cancer survival. Obstet Gynecol..

[41] Matusewicz L, Meissner J, Toporkiewicz M, Sikorski AF (2015). The effect of statins on cancer cells--review. Tumour biology : the journal of the International Society for Oncodevelopmental Biology and Medicine..

[42] Borgquist S, Bjarnadottir O, Kimbung S, Ahern TP (2018). Statins: a role in breast cancer therapy?. J Intern Med..

[43] Telfah M, Iwakuma T, Bur A, Shnayder L, Tsue T, Al-Kasspooles MM (2019). A window of opportunity trial of atorvastatin in p53-mutant and p53 wild type malignancies. J Clin Oncol..

[44] Verpaalen B, Neyts J, Delang L (2014). Are statins a viable option for the treatment of infections with the hepatitis C virus?. Antiviral Res..

[45] Bryan-Marrugo OL, Arellanos-Soto D, Rojas-Martinez A, Barrera-Saldana H, Ramos-Jimenez J, Vidaltamayo R (2016). The antidengue virus properties of statins may be associated with alterations in the cellular antiviral profile expression. Mol Med Rep.

[46] Drechsler H, Ayers C, Cutrell J, Maalouf N, Tebas P, Bedimo R (2017). Current use of statins reduces risk of HIV rebound on suppressive HAART. PLoS ONE..

[47] Marakasova ES, Eisenhaber B, Maurer-Stroh S, Eisenhaber F, Baranova A (2017). Prenylation of viral proteins by enzymes of the host: virus-driven rationale for therapy with statins and FT/GGT1 inhibitors. BioEssays..

[48] Shrivastava-Ranjan P, Flint M, Bergeron É, McElroy AK, Chatterjee P, Albariño CG (2018). Statins suppress ebola virus infectivity by interfering with glycoprotein processing. mBio.

[49] Biswas RR, Das MC, Rao ASRS, SRM K (2014). Effect of atorvastatin on memory in albino mice. J Clin Diagn Res.

[50] Lin F-C, Chuang Y-S, Hsieh H-M, Lee T-C, Chiu K-F, Liu C-K (2015). Early statin use and the progression of Alzheimer disease: a total population-based case-control study. Medicine..

[51] Saeedi Saravi SS, Saeedi Saravi SS, Arefidoust A, Dehpour AR (2017). The beneficial effects of HMG-CoA reductase inhibitors in the processes of neurodegeneration. Metab Brain Dis..

[52] McFarland AJ, Davey AK, McDermott CM, Grant GD, Lewohl J, Anoopkumar-Dukie S (2018). Differences in statin associated neuroprotection corresponds with either decreased production of IL-1β or TNF-α in an in vitro model of neuroinflammation-induced neurodegeneration. Toxicol Appl Pharmacol..

[53] Li H-H, Lin C-L, Huang C-N (2018). Neuroprotective effects of statins against amyloid β-induced neurotoxicity. Neural Regen Res..

[54] Anna L, Antonina G, Maria Giovanna M, Salvatore P, Salvatore P, Maurizio A (2018). Liver and statins: a critical appraisal of the evidence. Curr Med Chem.

[55] Mohammad S, Nguyen H, Nguyen M, Abdel-Rasoul M, Nguyen V, Nguyen CD (2019). Pleiotropic effects of statins: untapped potential for statin pharmacotherapy. Current Vascular Pharmacology..

[56] Adams SP, Tsang M, Wright JM (2015). Lipid-lowering efficacy of atorvastatin. Cochrane Database Syst Rev.

[57] Lewis JS, Windhorst AD, Zeglis BM. Radiopharmaceutical chemistry: Springer International Publishing; 2019.

[58] Beyzavi MH, Mandal D, Strebl MG, Neumann CN, D’Amato EM, Chen J (2017). 18F-deoxyfluorination of phenols via Ru π-complexes. ACS Cent Sci..

[59] Strebl MG, Campbell AJ, Zhao W-N, Schroeder FA, Riley MM, Chindavong PS (2017). HDAC6 brain mapping with [18F]bavarostat enabled by a Ru-mediated deoxyfluorination. ACS Cent Sci..

[60] Rickmeier J, Ritter T (2018). Site-specific deoxyfluorination of small peptides with [18F]fluoride. Angew Chem Int Ed..

[61] Baumann KL, Butler DE, Deering CF, Mennen KE, Millar A, Nanninga TN (1992). The convergent synthesis of CI-981, an optically active, highly potent, tissue selective inhibitor of HMG-CoA reductase. Tetrahedron Lett..

[62] Sagyam RR, Padi PR, Ghanta MR, Vurimidi H (2007). An efficient synthesis of highly substituted pyrrole and bis pyrrole derivatives. J Heterocycl Chem.

[63] Ballinger JR, Koziorowski J, Khalil MM (2017). Regulation of PET radiopharmaceuticals production in Europe. Basic science of PET imaging.

[64] Brittain HG. Profiles of drug substances, excipients and related methodology: Elsevier Science; 2010. 10.1016/S1871-5125(10)35013-8 22469228

[65] Ness GC, Gertz KR (2004). Hepatic HMG-CoA Reductase expression and resistance to dietary cholesterol. Exp Biol Med.

[66] Lagor WR, Heller R, De Groh ED, Ness GC (2007). Functional analysis of the hepatic HMG-CoA reductase promoter by in vivo electroporation. Exp Biol Med.

[67] Wu N, Sarna LK, Hwang S, Zhu Q, Wang P, Siow YL (2013). Regulation of Hmg-Coa reductase in diet-induced non-alcoholic fatty liver disease. Canadian J Cardiol.

[68] Keller GA, Barton MC, Shapiro DJ, Singer SJ (1985). 3-Hydroxy-3-methylglutaryl-coenzyme A reductase is present in peroxisomes in normal rat liver cells. PNAS..

[69] Rezvan A, Sur S, Jo H (2015). Novel animal models of atherosclerosis. Biomedical Engineering Letters..

[70] Wei S, Zhang Y, Su L, He K, Wang Q, Zhang Y (2015). Apolipoprotein E-deficient rats develop atherosclerotic plaques in partially ligated carotid arteries. Atherosclerosis..

[71] Sijbesma J, van Waarde A, Kristensen S, Kion I, Tietge UJF, Hillebrands JL (2019). OP-655: Characterization of the apolipoprotein E-deficient rat as novel model for atherosclerosis imaging. Eur J Nucl Med Mol Imaging..

[72] The Human Protein Atlas. https://www.proteinatlas.org/ENSG00000113161-HMGCR/tissue. Accessed 02 February 2020.

[73] Zarganes-Tzitzikas T, Neochoritis CG, Dömling A (2019). Atorvastatin (Lipitor) by MCR. ACS Med Chem Lett..

[74] Istvan ES, Deisenhofer J (2001). Structural mechanism for statin inhibition of HMG-CoA reductase. Science..

[75] Lee CW, Park C-S, Hwang I, Kim Y, Park D-W, Kang S-J (2011). Expression of HMG-CoA reductase in human coronary atherosclerotic plaques and relationship to plaque destabilisation. Heart..

