# Supplementary material for: [18F]Atorvastatin: synthesis of a potential molecular imaging tool for the assessment of statin-related mechanisms of action
Source: EJNMMI Res. 2020 Apr 15;10:34. doi: 10.1186/s13550-020-00622-4 (PMC7158976; doi:10.1186/s13550-020-00622-4)
Supplement: Supplementary file 1 — Additional file 1:. Supporting Information file. [file 13550_2020_622_MOESM1_ESM.pdf]

[^{18}F]Atorvastatin: synthesis of a potential molecular imaging tool for the assessment of statin-related mechanisms of action

Gonçalo S. Clemente[1], Jens Rickmeier[2], Inês F. Antunes[1], Tryfon Zarganes-Tzitzikas[3], Alexander Dömling[3], Tobias Ritter[2], Philip H. Elsinga[1†]

[1]Department of Nuclear Medicine and Molecular Imaging – University Medical Center Groningen, University of Groningen, The Netherlands; †p.h.elsinga@umcg.nl

[2]Max-Planck-Institut für Kohlenforschung, Germany

[3]Department of Drug Design, University of Groningen, The Netherlands

TABLE OF CONTENTS

Materials and methods **2**

Spectroscopy and instruments 2

Experimental procedures and compound characterization **3**

Benzyl ether pyrrole intermediate **6** 3

Hydroxy derivative intermediate **7** 4

(Cp)Ruthenium labeling precursor **8** 5

Atorvastatin protected precursor **11** 6

Atorvastatin (**12**) 7

Characterization of products (^{1}H NMR and ^{13}C NMR) **8**

Characterization of [^{18}F]atorvastatin (radio-HPLC and radio-TLC) **15**

Data from autoradiographic experiments **17**

Materials and methods

All substrates, reagents and solvents were purchased from commercial suppliers and used as received without any purification unless otherwise stated. Air- and moisture-sensitive manipulations were performed using oven-dried glassware under an atmosphere of argon or nitrogen. Air- and moisture-insensitive reactions were carried out under ambient atmosphere and monitored by thin-layer chromatography (TLC) or liquid chromatography-mass spectrometry (LC-MS). Thin-layer chromatography was performed on pre-coated silica gel 60 F_{254} plates and visualized by fluorescence quenching under UV light. Flash chromatography purifications were performed using commercial normal-phase silica gel (40–63 µm particle size). Concentration under reduced pressure was performed by rotary evaporation at 23–40 °C at an appropriate pressure. Purified compounds were further dried under vacuum (10^{-6}–10^{-3} bar). Yields refer to purified and spectroscopically pure compounds.

All of the procedures involving the handling of radioactive substances were carried out in a radiochemistry laboratory with the standard required conditions of radiological protection and safety. The use of personal protective equipment and lead shielding, with appropriate thickness to the manipulated activities, was equally transversal to all experimental radiochemistry procedures. Aqueous [^{18}F]fluoride used in this work was produced by the ^{18}O(*p*,*n*)^{18}F nuclear reaction in an IBA (Louvain-la-Neuve, Belgium) Cyclone 18/9 cyclotron. Manual radiolabeling was performed in radiochemistry fume hoods at negative air pressure with respect to the laboratory. Automated radiosynthesis was performed using a Synthra RNplus radiosynthesizer (Synthra GmbH) inside a hot cell at a negative air pressure with respect to the laboratory and remotely controlled by an external computer. Radiolabeled products were monitored and identified by radio-TLC and radio-HPLC.

Spectroscopy and instruments

High-resolution mass spectra (HRMS) were obtained using an electrospray ionization mass spectra (ESI) or electron ionization (EI) system from Waters Investigator Semi-prep 15 SFC-MS instrument and from Thermo Fisher Q Exactive Plus. High-performance liquid chromatography (HPLC) spectra were acquired in a Waters system using a 1525 binary HPLC pump, a 2489 UV/visible detector and a Berthold Technologies Flowstar LB 513 radio flow detector. Ultra-high performance liquid chromatography (UPLC) spectra were acquired using a Waters Acquity UPLC integrated system coupled to a Berthold Technologies Flowstar LB 513 radio flow detector. HPLC and UPLC data were processed with Waters Empower 3 software. Radio-TLC's and the sections used for autoradiography were scanned using a Perkin Elmer Packard Cyclone storage phosphor system and the acquired data analyzed with the OptiQuant 03.00 software.

Nuclear magnetic resonance (NMR) spectra were recorded on a Bruker 500 spectrometer operating at 500 MHz and 126 MHz for ^{1}H and ^{13}C acquisitions, respectively. Chemical shifts (δ) are reported in ppm, with the solvent residual peak as the internal standard, and coupling constants in hertz (Hz). The following abbreviations were used for spin multiplicity: s = singlet, d = doublet, t = triplet, dd = double doublet, m = multiplet, bs = broad singlet, h = heptete.

Experimental procedures and compound characterization

Benzyl ether pyrrole intermediate 6, *tert*-butyl 2-((4R,6R)-6-(2-(2-(4-(benzyloxy)phenyl)-5-isopropyl-3-phenyl-4-(phenylcarbamoyl)-1H-pyrrol-1-yl)ethyl)-2,2-dimethyl-1,3-dioxan-4-yl)acetate

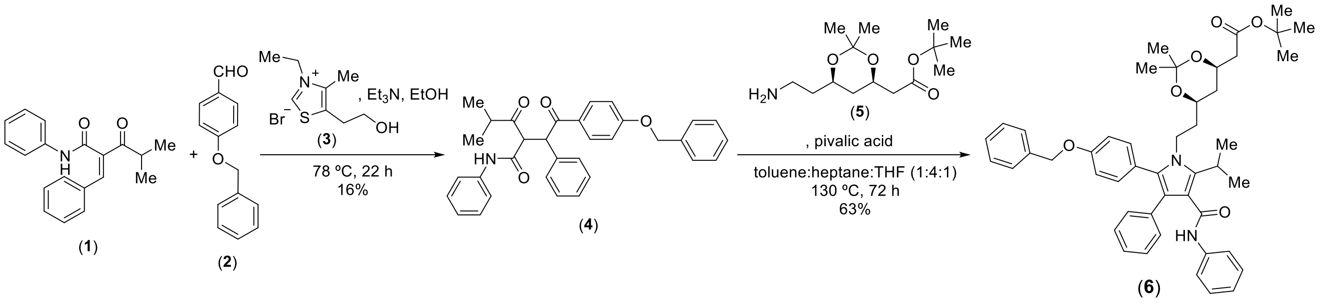

A mixture of 2-benzylidene-N-phenyl-isobutyloyl acetamide (**1**, 5.00 g, 17.0 mmol, 1.00 equiv.), 3-ethyl-5-(2-hydroxyethyl)-4-methyl-3-thiazolium bromide (**3**, 0.860 g, 3.41 mmol, 0.200 equiv.), triethylamine (2.61 mL, 1.90 g, 18.8 mmol, 1.10 equiv.), 4-benzyloxybenzaldehyde (**2**, 3.98 g, 18.8 mmol, 1.10 equiv.) and and absolute ethanol (10 mL, 1.7 mol/L) was heated at reflux (78 °C) with vigorous stirring for 15 h. At this time point, the second portion of 3-ethyl-5-(2-hydroxyethyl)-4-methylthiazolium bromide (**3**, 0.860 g, 3.41 mmol, 0.200 equiv.) was added, and the reaction mixture was stirred and heated at reflux for additional 7 hours. Isopropyl alcohol (35 mL) was added to the reaction mixture at 78 °C, and the oil bath was removed. After 12 hours the mixture was filtered and dried in vacuum to afford a colorless solid in 16% yield (**4**, 1.38 g, 2.72 mmol).

^{1}H NMR of **4** (500 MHz, DMSO-d_6) δ: 10.15 (s, 1H), 8.05–7.97 (m, 2H'), 7.45–7.39 (m, 2H), 7.41–7.37 (m, 2H), 7.38–7.32 (m, 3H), 7.32–7.27 (m, 2H), 7.26–7.17 (m, 4H), 7.17–7.08 (m, 1H), 7.08–7.04 (m, 2H), 7.05–6.96 (m, 1H), 5.40 (d, *J* = 11.0 Hz, 1H), 5.17 (s, 2H), 4.85 (d, *J* = 10.9 Hz, 1H), 2.90 (h, *J* = 6.9 Hz, 1H), 1.16 (d, *J* = 7.0 Hz, 3H), 0.94 (d, *J* = 6.6 Hz, 3H).

^{13}C NMR of **4** (126 MHz, DMSO-d_6) δ: 207.9, 196.1, 165.1, 162.2, 138.1, 136.4, 135.8, 131.1, 128.8, 128.6, 128.5, 128.0, 127.8, 127.4, 123.9, 119.6, 114.7, 69.5, 62.9, 51.6, 18.8, 18.0.

HRMS-EI of **4**: m/z *calcd.* for $C_{33}H_{31}N_1O_4Na$ $[M+Na]^+$ 528.214528; *found* 528.215034.

To a solution of the previously synthesized 2-(2-(4-(benzyloxy)phenyl)-2-oxo-1-phenylethyl)-4-methyl-3-oxo-N-phenylpentanamide (**4**, 1.27 g, 2.51 mmol, 1.00 equiv.) and tert-butyl 2-((4R,6R)-6-(2-aminoethyl)-2,2-dimethyl-1,3-dioxan-4-yl)acetate (**5**, 0.891 g, 3.26 mmol, 1.30 equiv.) in toluene:heptane:tetrahydrofuran (1:4:1 v:v) (42 mL) was added pivalic acid (0.576 mL, 5.01 mmol, 2.00 equiv.) under nitrogen atmosphere. The reaction mixture was stirred at 130 °C for 72 h. The solvent was removed under vacuum and the residue was purified by silica flash chromatography (Hex:EtOAc). The desired product **6** was obtained as a white solid in 63% yield (1.17 g, 2. 51 mmol).

^{1}H NMR of **6** (500 MHz, $CDCl_3$) δ: 7.46–7.42 (m, 2H), 7.42–7.37 (m, 2H), 7.36–7.32 (m, 1H), 7.23–7.15 (m, 7H), 7.13 (d, *J* = 8.5 Hz, 2H), 7.09 (d, *J* = 8.5 Hz, 2H), 6.98 (t, *J* = 7.3 Hz, 1H), 6.92 (d, *J* = 8.2 Hz, 2H), 6.89 (s, 1H), 5.04 (s, 2H), 4.17 (dtd, *J* = 9.0, 6.5, 2.2 Hz, 1H), 4.09 (ddd, *J* = 15.2, 10.5, 5.1 Hz, 1H), 3.90–3.81 (m, 1H), 3.69 (td, *J* = 8.4, 7.8 Hz, 1H), 3.59 (p, *J* = 7.1 Hz, 1H), 2.39 (dd, *J* = 15.9, 7.1 Hz, 1H), 2.25 (dd, *J* = 15.2, 6.0 Hz, 1H), 1.79–1.62

(m, 2H), 1.60–1.56 (m, 1H) 1.55 (dd, *J* = 7.1, 2.2 Hz, 6H), 1.44 (s, 9H), 1.37 (s, 3H), 1.35–1.33 (m, 1H) 1.32 (s, 3H), 1.06 (dd, *J* = 11.9 Hz, 1H).

^{13}C NMR of **6** (126 MHz, $CDCl_3$) δ: 170.3, 165.1, 158.4, 141.3, 138.6, 136.8, 135.1, 132.8, 130.6, 129.8, 128.7, 128.6, 128.3, 128.1, 127.6, 126.4, 124.8, 123.5, 121.5, 119.6, 115.2, 114.6, 98.7, 80.7, 70.0, 66.6, 66.0, 42.6, 40.9, 38.2, 36.1, 30.0, 28.2, 26.2, 21.9, 21.7, 19.8.

HRMS-ESI of **6**: m/z *calcd.* for $C_{47}H_{54}N_2O_6Na$ $[M+Na]^+$ 765.387406, *found* 765.387990.

Hydroxy derivative intermediate 7, *tert*-butyl 2-((4R,6R)-6-(2-(2-(4-hydroxyphenyl)-5-isopropyl-3-phenyl-4-(phenylcarbamoyl)-1H-pyrrol-1-yl)ethyl)-2,2-dimethyl-1,3-dioxan-4-yl)acetate

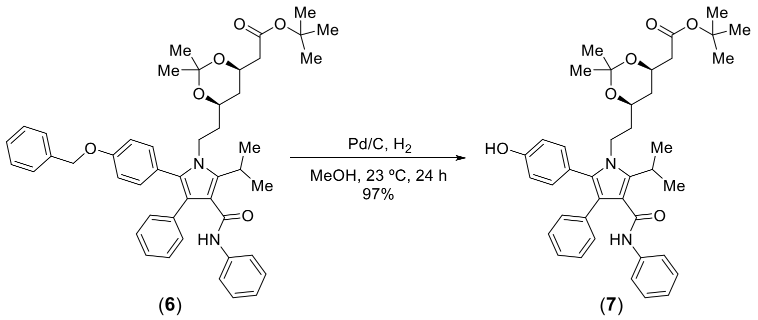

A round-bottom flask (100 mL) equipped with a septum and a magnetic stirring bar was charged with the previously formed intermediate **6** (765 mg, 1.03 mmol, 1.00 equiv.), palladium on carbon (10 w%, 110 mg, 103 µmol, 0.100 equiv.), and methanol (50 mL). Argon was bubbled through the reaction mixture for 5 minutes while stirring. Afterward, dihydrogen was bubbled through the reaction mixture for 10 minutes while stirring. The reaction mixture was stirred under a dihydrogen atmosphere at 23 °C for 24 h. Argon was bubbled through the reaction mixture for 5 minutes while stirring. The reaction mixture was filtered through a Celite plug, and the Celite was washed with methanol (2 x 5 mL). The filtrate was concentrated under vacuum. A round-bottom flask (10 mL) equipped with a septum and a magnetic stirring bar was charged with pale yellow filtrate, *p*-toluenesulfonic acid (1.96 mg, 10.3 µmol, 1.00 mol%), 2,2-dimethoxypropan (190 µL, 161 mg, 1.54 mmol, 1.50 equiv.), and acetone (2 mL). The reaction mixture was stirred at 23 °C for 2 hours. The resulting suspension was diluted with dichloromethane (20 mL), and the solution was washed with saturated aqueous sodium bicarbonate solution (10 mL) and saturated aqueous sodium chloride solution. The organic layer was dried over sodium sulfate, filtered, and concentrated under vacuum to dryness to afford **7** in 97% yield as a pale yellow powder (653 mg, 1.00 mmol).

^{1}H NMR of **7** (500 MHz, $CDCl_3$) δ: 7.21–7.10 (m, 7H), 7.06 (d, *J* = 7.9 Hz, 2H), 7.03 (d, *J* = 8.4 Hz, 2H), 6.98 (t, *J* = 7.3 Hz, 1H), 6.91 (s, 1H), 6.77 (d, *J* = 8.3 Hz, 2H), 6.43 (bs, 1H), 4.17–4.10 (m, 1H), 4.05 (ddd, *J* = 15.1, 10.3, 5.3 Hz, 1H), 3.82 (ddd, *J* = 14.9, 10.0, 5.6 Hz, 1H), 3.66 (tt, *J* = 11.1, 5.3 Hz, 1H), 3.57 (p, *J* = 7.1 Hz, 1H), 2.37 (dd, *J* = 15.1, 7.2 Hz, 1H), 2.23 (dd, *J* = 15.1, 5.8 Hz, 1H), 1.72–1.62 (m, 2H), 1.51 (dd, *J* = 7.1, 4.9 Hz, 6H), 1.44 (s, 9H), 1.35 (s, 3H), 1.31 (s, 3H), 1.29–1.22 (m, 1H) 1.00 (dd, *J* = 11.9 Hz, 1H).

^{13}C NMR of **7** (126 MHz, $CDCl_3$) δ: 170.7, 165.5, 156.0, 141.2, 138.4, 135.0, 132.9, 130.6, 130.0 ,128.8, 128.4, 126.4, 124.1, 123.8, 121.4, 119.9, 115.6, 115.1, 98.8, 81.1, 66.1, 66.3, 42.7, 40.8, 38.1, 36.0, 30.0, 28.2, 26.2, 21.9, 21.8, 19.8.

HRMS-ESI of **7**: m/z *calcd.* for $C_{40}H_{48}N_2O_6Na$ $[M+Na]^+$ 675.340456, *found* 675.341080.

(Cp)Ruthenium labeling precursor (8)

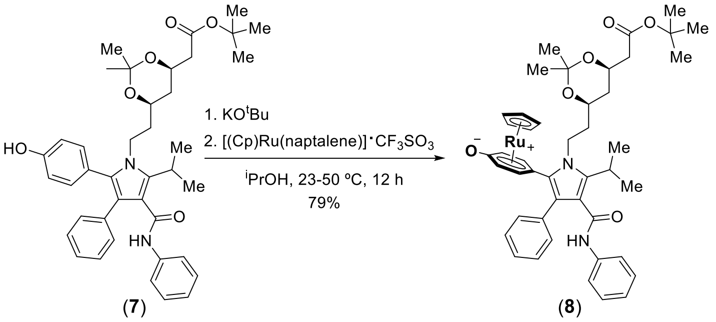

(7) (8)

Under an inert atmosphere, an oven-dried round-bottom flask (50 mL) equipped with a Teflon-coated egg-shaped magnetic stirring bar was charged with **7** (628 mg, 962 µmol, 1.00 equiv.), potassium *tert*-butoxide (113 mg, 1:01 mmol, 1.05 equiv.), and isopropyl alcohol (20 mL). The solution was degassed by bubbling argon through it for 20 minutes while stirring. To the solution was added [(Cp)Ru(η^6-naphthalene)]·CF_3SO_3 (512 mg, 1.15 mmol, 1.20 equiv.) and the resulting orange solution was stirred at 50 °C for 12 hours. The reaction mixture was concentrated to dryness under reduced pressure. The brown residue was dissolved in dichloromethane (20 mL), and the solution was washed with water (10 mL) and saturated aqueous sodium chloride solution (10 mL). The organic layer was dried over sodium sulfate, filtered, and concentrated to dryness under vacuum. The brown residue was purified by HPLC on an YMC-Actus Triart C18 column ((30×150 mm, 5 µm + 30×50 mm, 5 µm), flow rate = 42.5 mL/min., 35 °C) with a linear gradient from 40:60 (0.1% TFA in H_2O:MeOH, v:v) to 03:97 (0.1% TFA in H_2O:MeOH, v:v) over 8 minutes. The collected fractions containing the product (t ≈ 7.5 min.) were combined, diluted with 100 mL saturated aqueous sodium chloride solution, basified to pH 10 with saturated aqueous sodium bicarbonate solution, and the resulting solution was concentrated by rotary evaporation (100 mbar, 35 °C) until no more methanol was evaporated. The suspension was extracted with dichloromethane (3 × 100 mL), and the combined organic layers were dried over sodium sulfate, filtered, and concentrated in vacuum to dryness to afford the labeling precursor **8** as a beige powder in 79% yield (620 mg, 758 µmol).

^{1}H NMR of **8** (400 MHz, DMSO-*d*6) δ: 9.76 (s, 1H), 7.48–7.43 (m, 2H), 7.27–7.14 (m, 7H), 7.01–6.96 (m, 1H), 5.51–5.45 (m, 1H), 5.21 (dd, *J* = 6.9, 1.8 Hz, 1H), 4.92 (dd, *J* = 6.9, 2.9 Hz, 1H), 4.90 (s, 5H), 4.79 (dd, *J* = 6.8, 2.0 Hz, 1H), 4.49–4.39 (m, 1H), 4.32–4.23 (m, 1H), 4.16–4.02 (m, 2H), 3.18 (p, *J* = 7.0 Hz, 1H), 2.43 (dd, *J* = 15.2, 4.8 Hz, 1H), 2.27 (dd, *J* = 15.2, 8.1 Hz, 1H), 2.0–1.9 (m, 1H), 1.82–1.71 (m, 1H), 1.64 (dt, *J* = 12.7, 2.5 Hz, 1H), 1.49 (s, 3H), 1.42 (s, 9H), 1.40–1.32 (m, 9H), 1.27–1.18 (m, 1H).

^{13}C NMR of **8** (101 MHz, DMSO-*d*6) δ: 169.6, 165.6, 155.9, 139.3, 137.6, 134.8, 130.1, 128.4, 127.9, 126.2, 123.9, 123.0, 121.2, 119.4, 118.6, 98.2, 87.2, 86.1, 85.3, 79.8, 76.7, 71.8, 71.7, 66.4, 65.8, 42.1, 38.7, 35.4, 30.0, 27.8, 25.5, 22.8, 21.7, 20.0.

HRMS-EI of **8**: m/z *calcd.* for $C_{45}H_{53}N_2O_6Ru$ [M-OTf]$^+$ 819.29416, *found* 819.295570.

Atorvastatin protected precursor, *tert*-butyl 2-[(4R,6R)-6-[2-[2-(4-fluorophenyl)-5-isopropyl-3-phenyl-4-(phenylcarbamoyl)-1H-pyrrol-1-yl]ethyl]-2,2-dimethyl-1,3-dioxan-4-yl]acetate (11)

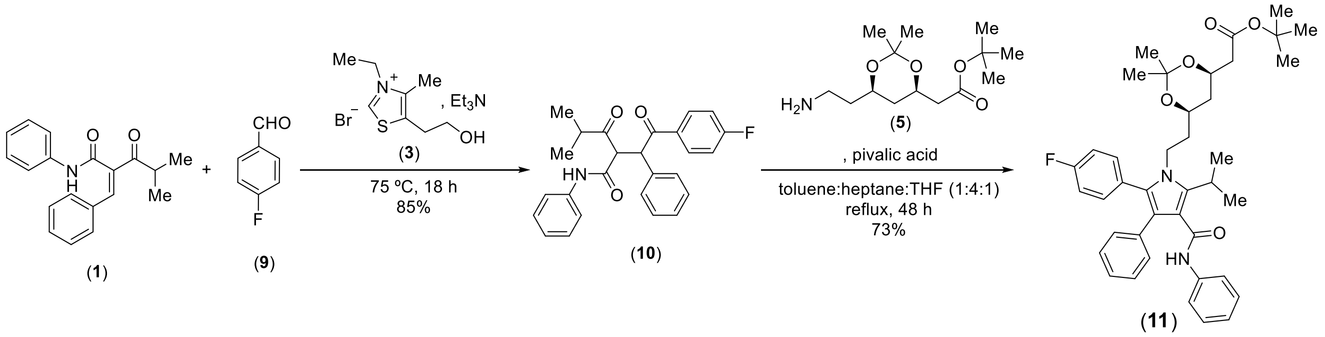

A mixture of 2-benzylidene-N-phenyl-isobutyloyl acetamide (**1**, 10.0 g, 34.0 mmol, 1.00 equiv.), 3-ethyl-5-(2-hydroxyethyl)-4-methyl-3-thiazolium bromide (**3**, 1.72 g, 6.80 mmol, 0.20 equiv.), triethylamine (10.5 mL, 75.0 mmol, 2.20 equiv.) and 4-fluorobenzaldehyde (**9**, 5.60 g, 45.0 mmol, 1.32 equiv.) was heated at 75 °C under argon atmosphere with vigorous stirring for 16 h. The reaction was monitored by TLC until the consumption of the N-phenylpentanamide was achieved. Isopropyl alcohol (50 mL) was added, and the reaction mixture was maintained at 25 °C for 4 hours under stirring. The remaining solid was vacuum filtered and washed with 55 mL of water followed by 40 mL of isopropyl alcohol. The product was dried under high vacuum for 4 hours, affording a white crystalline solid **10** in 85% yield (12.1 g, 29.0 mmol).

^{1}H NMR of **10** (250 MHz, DMSO-*d6*) δ: 0.94 (d, *J* = 6.7 Hz, 3H), 1.17 (d, *J* = 7.0 Hz, 3H), 2.88-2.93 (m, 1H), 4.87 (d, *J* = 11.0 Hz, 1H), 5.42 (d, *J* = 11.0 Hz, 1H), 7.02 (t, *J* = 7.3 Hz, 1H), 7.14 (t, *J* = 7.3 Hz, 1H), 7.23 (q, *J* = 8.0 Hz, 4H), 7.30 (t, *J* = 8.6 Hz, 4H), 7.36 (d, *J* = 7.4 Hz, 2H) 8.13 (dd, *J* = 8.7, 5.5 Hz, 2H), 10.18 (s, 1H).

^{13}C NMR of **10** (126 MHz, DMSO-*d6*) δ: 208.1, 196.4, 165.6 (d, J = 253.3 Hz), 165.0 138.1, 135.1, 132.19 (d, J = 2.5 Hz), 131.76 (d, J = 9.3 Hz), 128.9, 128.7, 128.6, 127.6, 124.0, 119.7, 115.85 (d, J = 21.9 Hz), 63.0, 51.8, 38.9, 18.8, 17.9.

HRMS-ESI of **10**: m/z *calcd.* for $C_{26}H_{25}F_1N_1O_3$ $[M+H]^+$ 418.1819; *found* 418.1914.

To a solution of the previously synthesized 2-[2-(4-fluorophenyl)-2-oxo-1-phenylethyl]-4-methyl-3-oxo-N-phenylpentanamide (**10**, 8.40 g, 20.1 mmol, 1.00 equiv.) and *tert*-butyl 2-((4R,6R)-6-(2-aminoethyl)-2,2-dimethyl-1,3-dioxan-4-yl)acetate (7.40 g, 27.1 mmol, 1.35 equiv.) in toluene:heptane:tetrahydrofuran (1:4:1 v:v) (156 mL) was added pivalic acid (4.00 g, 39.4 mmol, 1.96 equiv.) under nitrogen atmosphere. The reaction mixture was refluxed for 24 hours with azeotropic removal of water, monitored by TLC, cooled to room temperature and extracted with ethyl acetate (3 x 700 mL). The organic phase was washed with a saturated aqueous sodium chloride solution (500 mL). The solvent was removed under vacuum, the desired product was

obtained **11** as a pale yellow solid in 73% yield (9.60 g, 14.7 mmol) after purification by column chromatography (PE:EtOAc).

^{1}H NMR of **11** (500 MHz, $CDCl_3$) δ: 7.22-7.13 (m, 8H), 7.07 (d, *J* = 7.9 Hz, 2H), 7.01-6.97 (m, 3H), 6.86 (s, 1H), 4.19-4.03 (m, 2H), 3.86-3.78 (m, 1H), 3.72-3.65 (m, 1H), 3.57 (dt, *J* = 14.3, 7.1 Hz, 1H), 2.38 (dd, *J* = 15.3, 6.9 Hz, 1H), 2.24 (dd, *J* = 15.3, 6.2 Hz, 1H), 1.70-1.63 (m, 2H), 1.59-1.56 (m, 2H), 1.53 (d, *J* = 7.1 Hz, 6H), 1.43 (s, 9H), 1.36 (s, 3H), 1.30 (s, 3H).

^{13}C NMR of **11** (126 MHz, $CDCl_3$) δ: 170.2, 164.8, 162.29 (d, *J* = 247.8 Hz), 141.5, 138.4, 134.7, 133.20 (d, *J* = 8.0 Hz), 130.5, 129.3, 128.8, 128.7, 128.4, 128.28 (d, *J* = 3.3 Hz), 126.6, 123.5, 121.8, 119.6, 115.37 (d, *J* = 21.4 Hz), 98.7, 80.7, 66.4, 65.9, 42.5, 40.9, 38.1, 36.0, 29.9, 28.1, 26.1, 21.8, 21.6, 19.7.

HRMS-ESI of **11**: m/z *calcd.* for $C_{40}H_{48}FN_2O_5$ $[M+H]^+$ 655.3547, *found* 655.3574.

Atorvastatin (12)

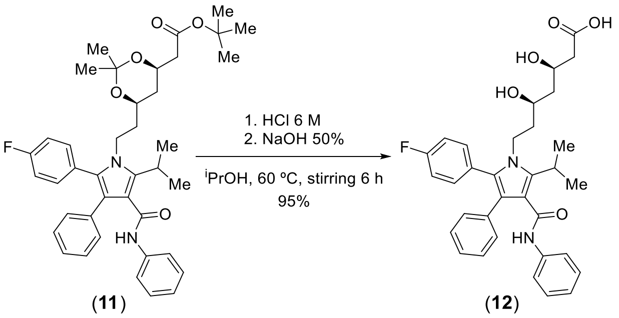

To the atorvastatin protected precursor **11** (1.00g, 1.53 mmol, 1.00 equiv.) was added 25 mL of a solution of aqueous hydrochloric acid 6 M in isopropyl alcohol (20:1 v:v). This solution was left under vigorous stirring at 60 °C for 3 h. Further addition of 3 mL aqueous sodium hydroxide 50% (w/w), under 60 °C and left to react for 3 hours, produced atorvastatin (**12**) in 95% yield after simple cooling, washing with abundant cold water and filtration (0.80 g, 1.43 mmol).

^{1}H NMR of **12** (500 MHz, DMSO-*d*6) δ: 9.86 (s, 1H), 7.52 (d, *J* = 7.8 Hz, 2H), 7.27 – 7.16 (m, 7H), 7.07 (d, *J* = 4.4 Hz, 4H), 6.99 (dd, *J* = 8.5, 3.6 Hz, 2H), 3.98 – 3.88 (m, 1H), 3.82 – 3.70 (m, 1H), 3.70 – 3.62 (m, 1H), 3.59 – 3.49 (m, 1H), 3.43 (d, *J* = 7.0 Hz, 1H), 3.28 – 3.19 (m, 1H), 2.00 (dd, *J* = 15.1, 4.1 Hz, 1H), 1.82 (dd, *J* = 15.1, 8.1 Hz, 1H), 1.60 (t, *J* = 12.4 Hz, 1H), 1.51 (dq, *J* = 11.4, 5.9 Hz, 1H), 1.36 (d, *J* = 6.1 Hz, 6H), 1.17 (d, *J* = 13.6 Hz, 1H), 1.10 (s, 1H), 1.05 (t, *J* = 7.0 Hz, 1H).

^{13}C NMR of **12** (126 MHz, DMSO-*d*6) δ: 176.4, 166.2, 162.6, 160.6, 139.5, 135.9, 135.0, 133.4, 129.2, 128.79, 128.76, 128.5, 127.7, 127.3, 125.4, 123.0, 120.6, 119.4, 117.5, 115.5, 115.3, 66.9, 66.4, 66.35, 66.28, 65.9, 56.1, 44.1, 43.7, 43.5, 25.74, 25.70, 22.3.

HRMS-ESI of **12**: m/z *calcd.* for $C_{33}H_{36}FN_2O_5$ $[M+H]^+$ 559.2608, *found* 559.2610.

Characterization of products (^{1}H NMR and ^{13}C NMR)

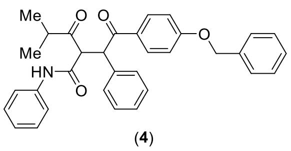

(**4**)

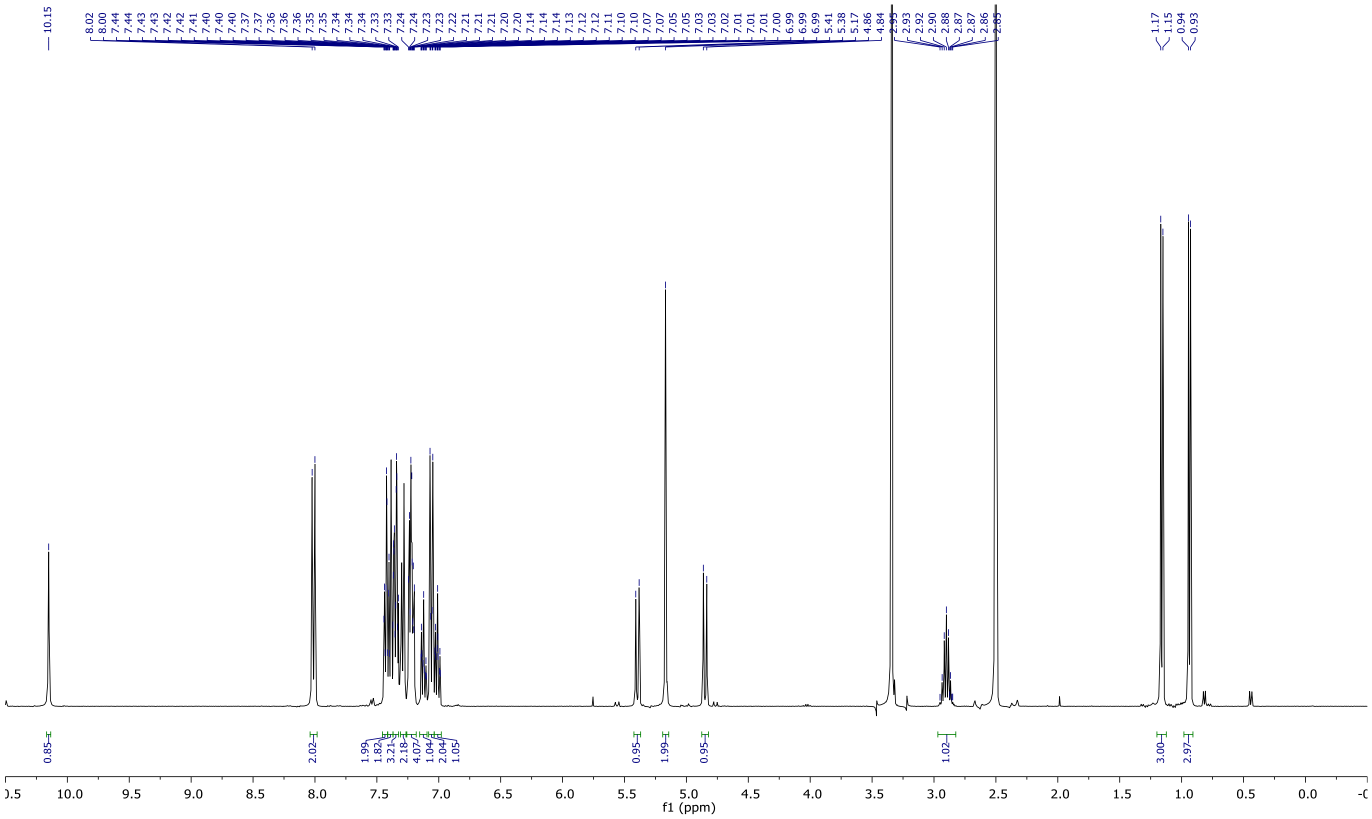

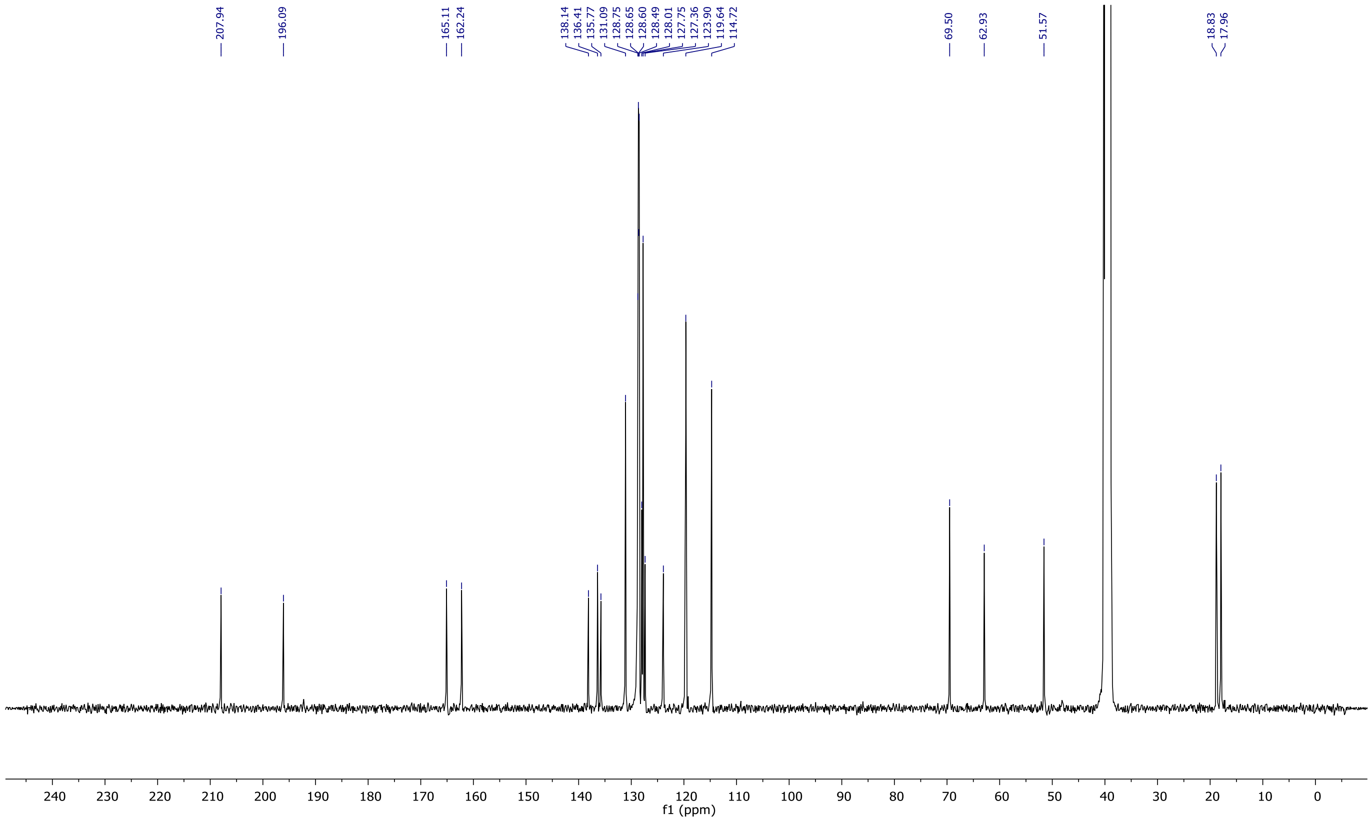

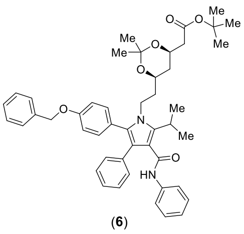
O
O
Me
Me
Me
Me
O
Me
O
O
N
Me
Me
O
HN

(**6**)

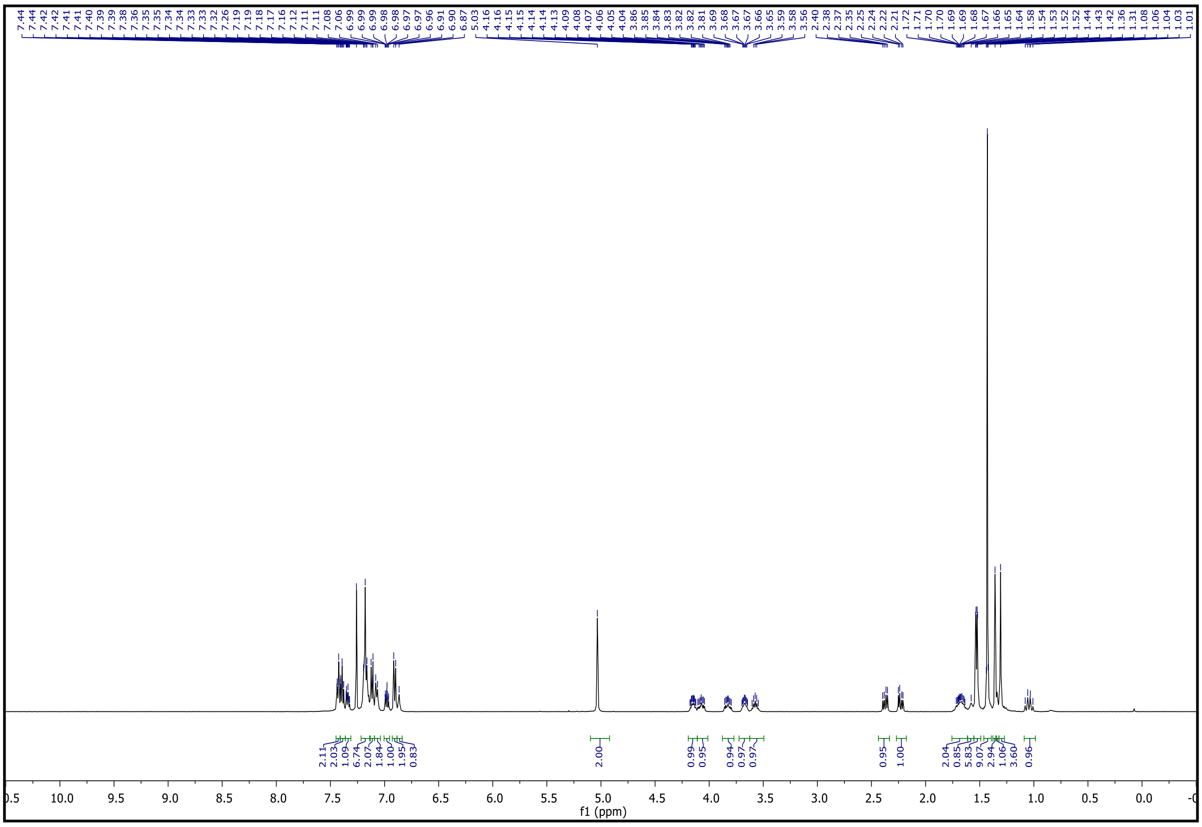
7.44 7.44 7.42 7.42 7.41 7.41 7.40 7.39 7.39 7.38 7.36 7.35 7.35 7.34 7.34 7.33 7.33 7.32 7.26 7.19 7.19 7.18 7.17 7.16 7.12 7.11 7.11 7.08 7.06 6.99 6.99 6.99 6.98 6.98 6.97 6.97 6.96 6.91 6.90 6.87 5.03 4.16 4.16 4.15 4.15 4.14 4.14 4.13 4.09 4.08 4.07 4.06 4.05 4.04 3.86 3.85 3.84 3.83 3.82 3.82 3.81 3.69 3.68 3.67 3.67 3.66 3.65 3.59 3.58 3.56 2.40 2.38 2.37 2.35 2.25 2.24 2.22 2.21 1.72 1.71 1.70 1.70 1.69 1.69 1.68 1.67 1.66 1.65 1.64 1.58 1.54 1.53 1.52 1.52 1.44 1.43 1.42 1.36 1.31 1.08 1.06 1.04 1.03 1.01
2.11 2.03 1.09 6.74 2.07 1.84 1.00 1.95 0.83
2.00
0.99 0.95
0.94 0.97 0.97
0.95 1.00
2.04 0.85 5.83 9.07 2.94 1.06 3.60
0.96
10.0 9.5 9.0 8.5 8.0 7.5 7.0 6.5 6.0 5.5 5.0 4.5 4.0 3.5 3.0 2.5 2.0 1.5 1.0 0.5 0.0
f1 (ppm)

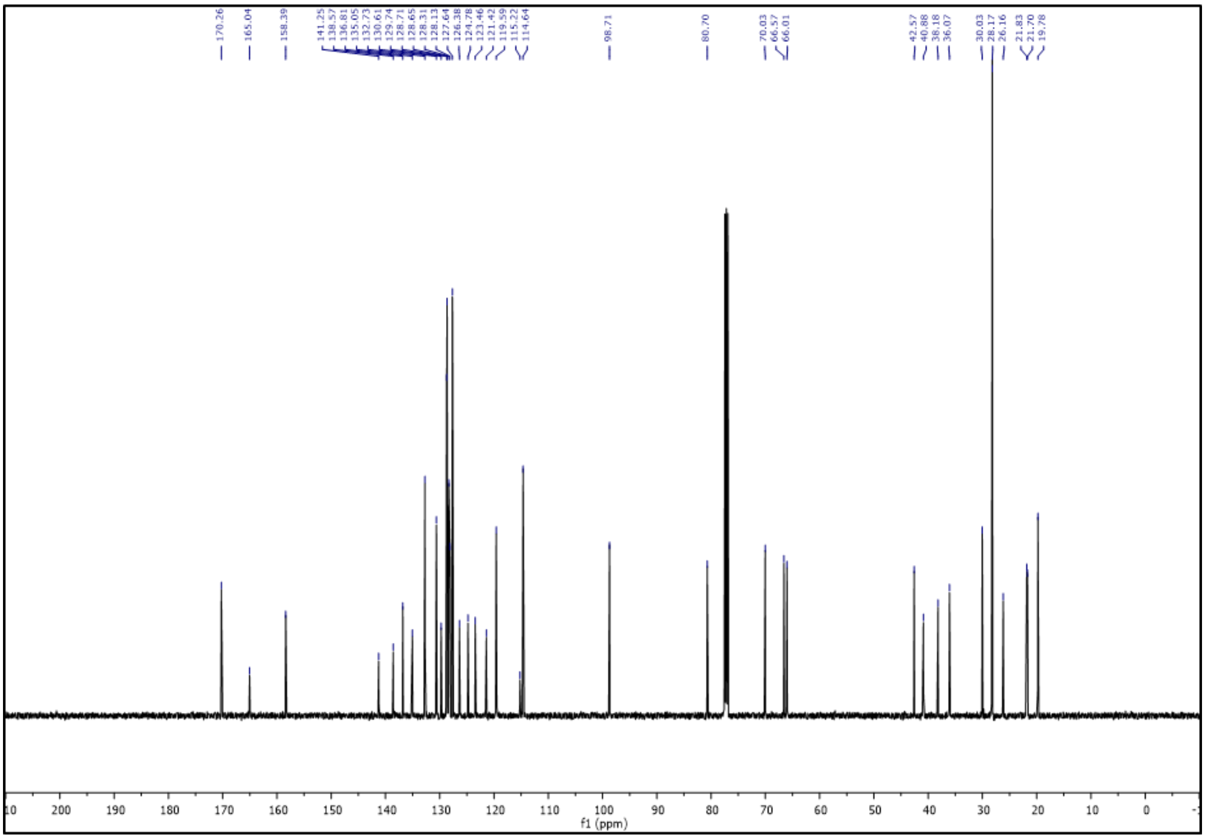
170.26
165.04
158.39
141.25 138.57 136.81 135.05 132.73 130.61 129.74 128.71 128.65 128.31 128.13 127.64 126.38 124.78 123.46 121.42 119.59 115.22 114.64
98.71
80.70
70.03 66.57 66.01
42.57 40.88 38.18 36.07
30.03 28.17 26.16
21.83 21.70 19.78
200 190 180 170 160 150 140 130 120 110 100 90 80 70 60 50 40 30 20 10 0
f1 (ppm)

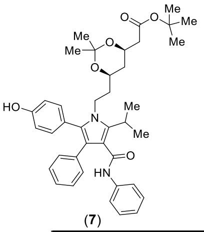

(7)

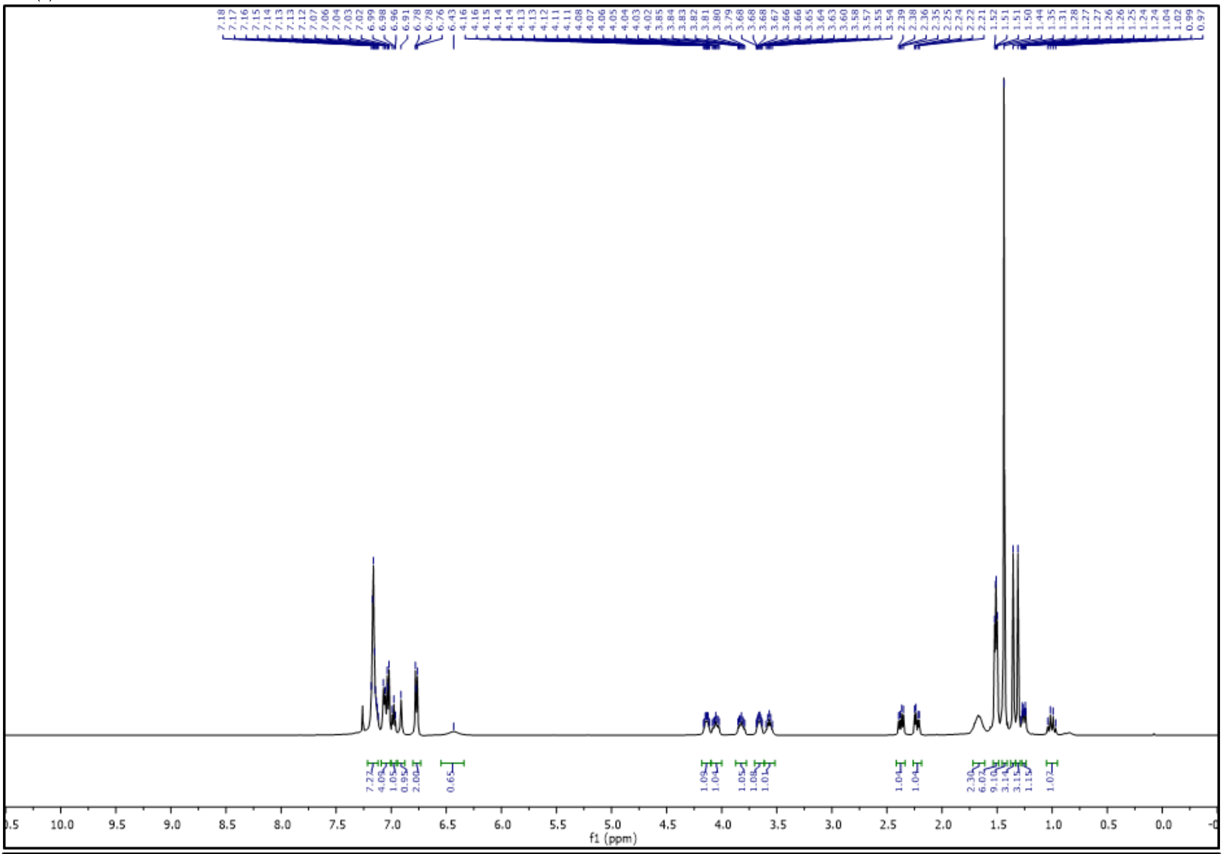

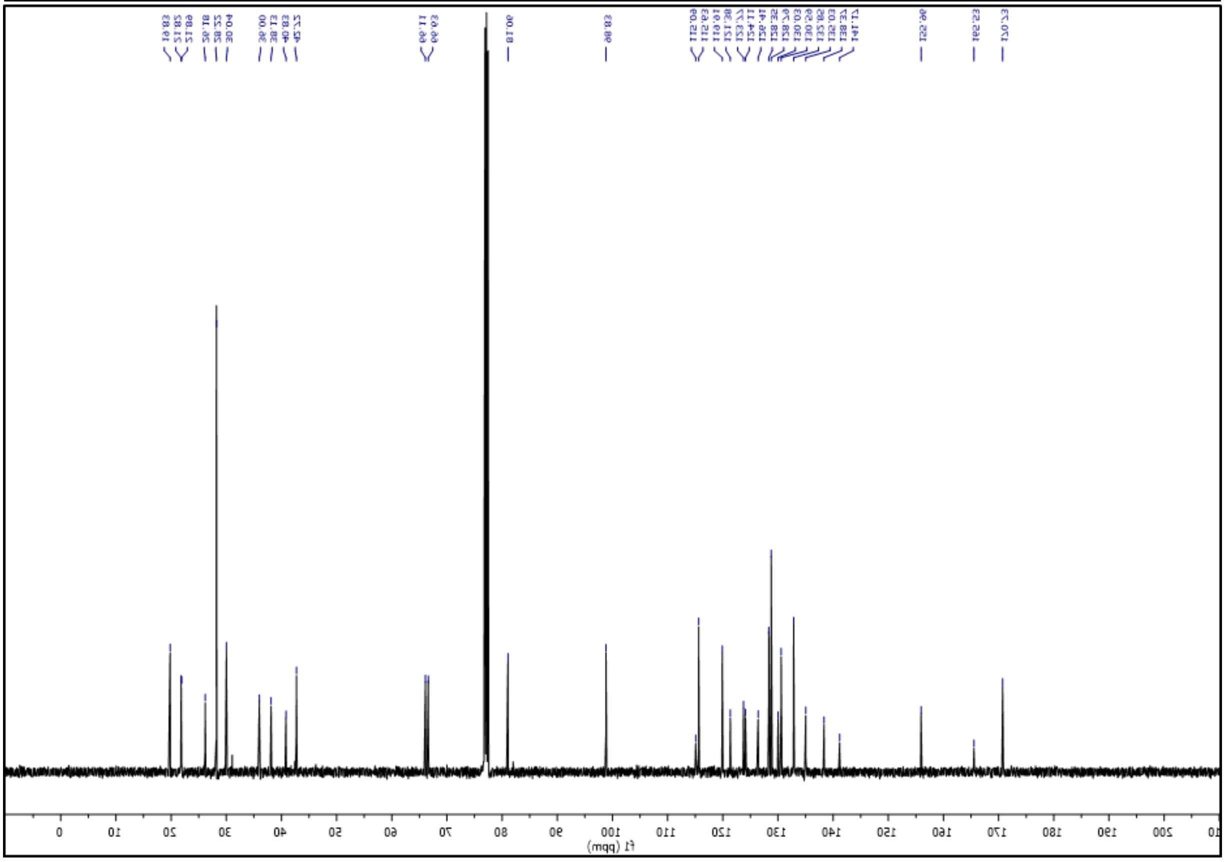

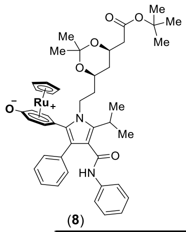

(**8**)

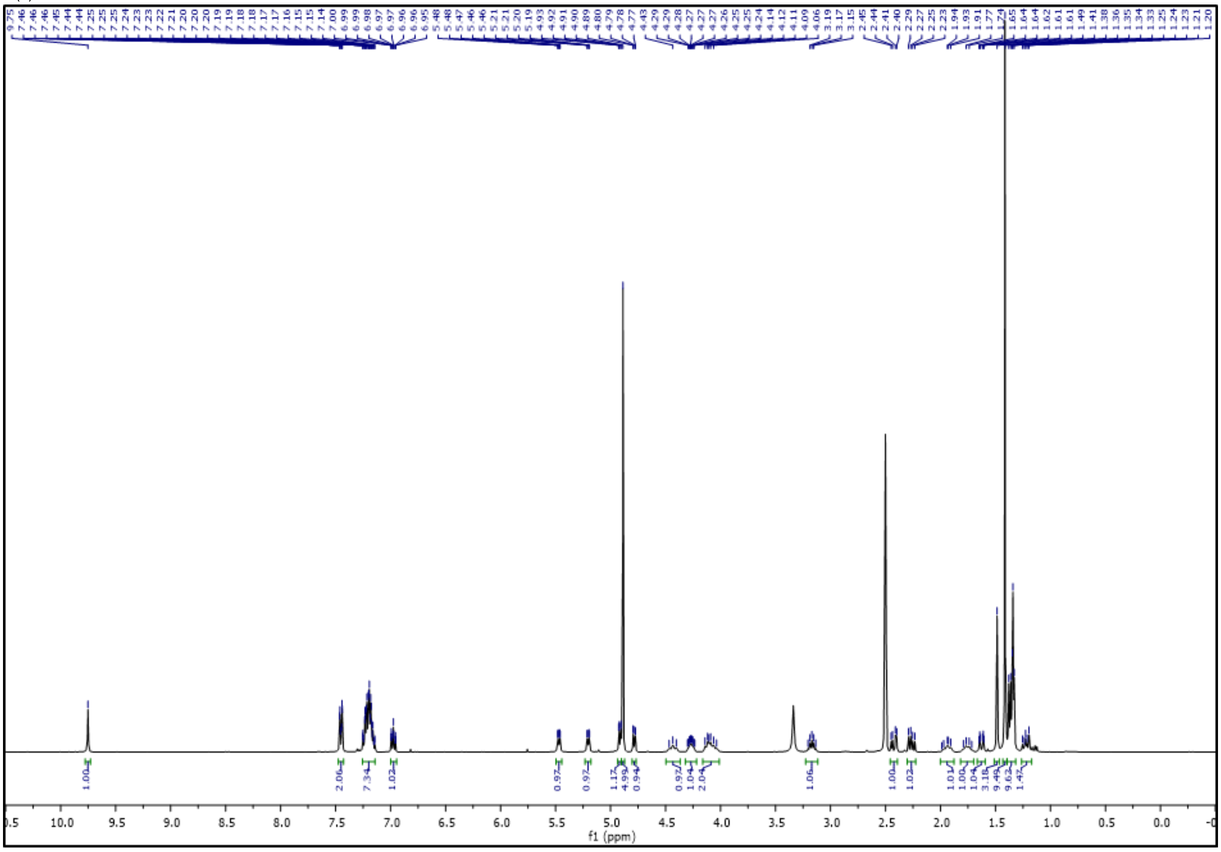

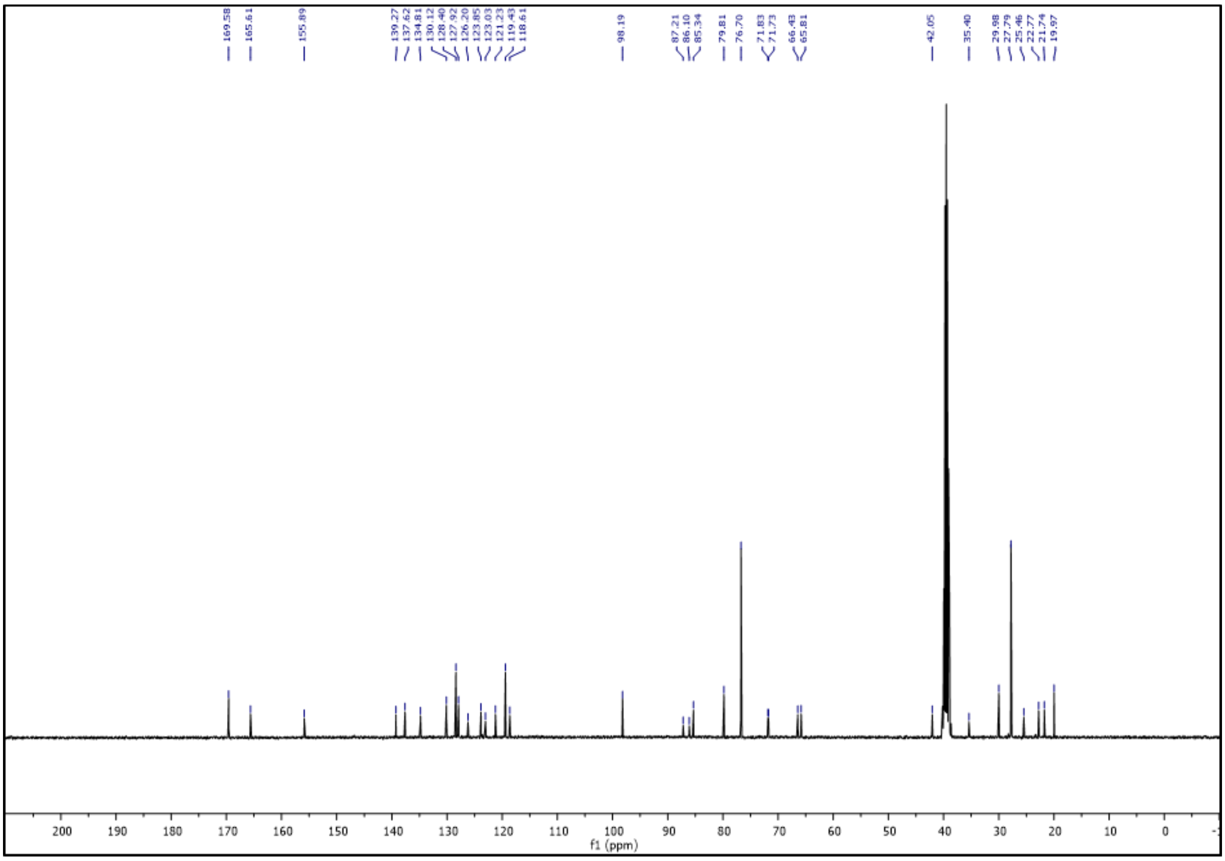

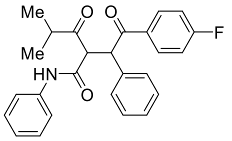
Me
O
O
F
Me
HN
O

(**10**)

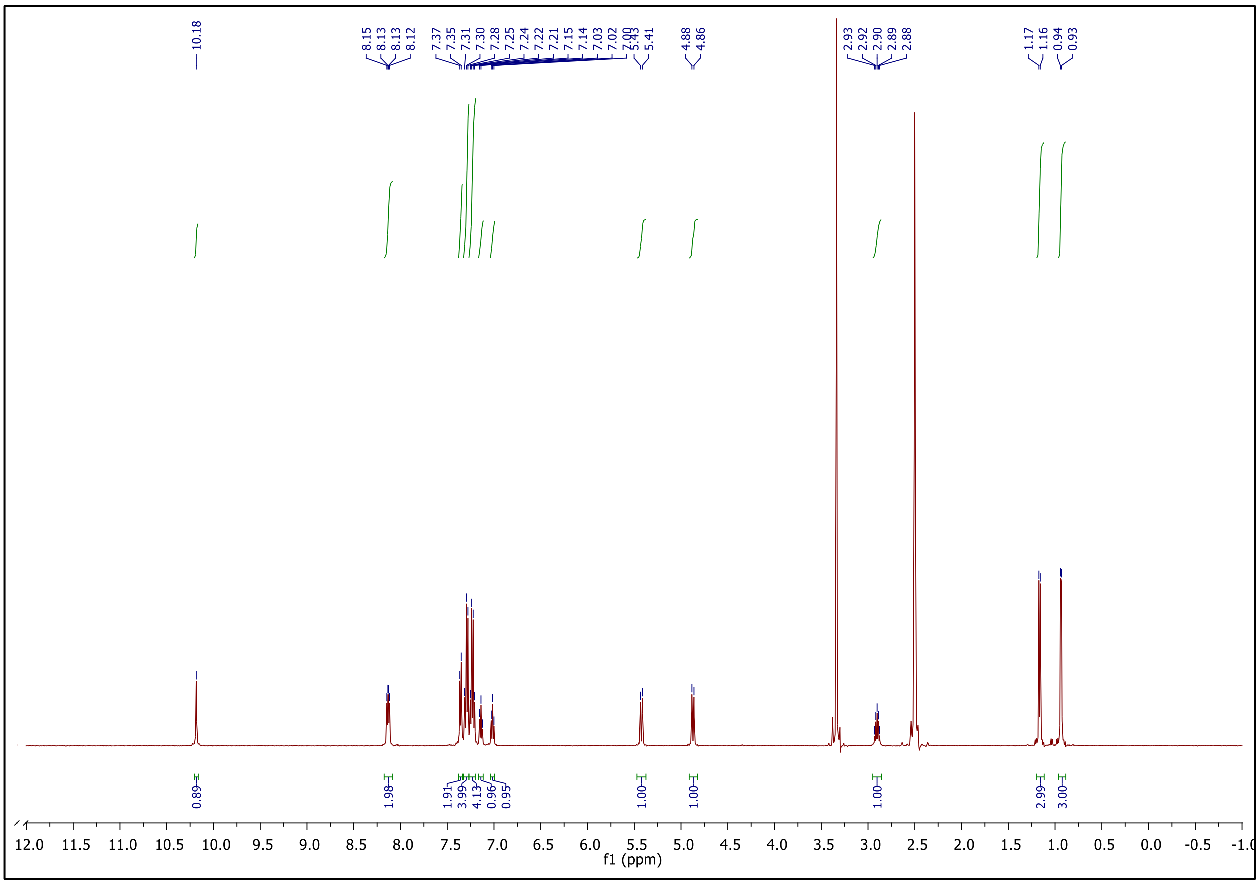
10.18
8.15
8.13
8.13
8.12
7.37
7.35
7.31
7.30
7.28
7.25
7.24
7.22
7.21
7.15
7.14
7.03
7.02
7.00
5.43
5.41
4.88
4.86
2.93
2.92
2.90
2.89
2.88
1.17
1.16
0.94
0.93
0.89
1.98
1.91
3.99
4.13
0.96
0.95
1.00
1.00
1.00
2.99
3.00
f1 (ppm)

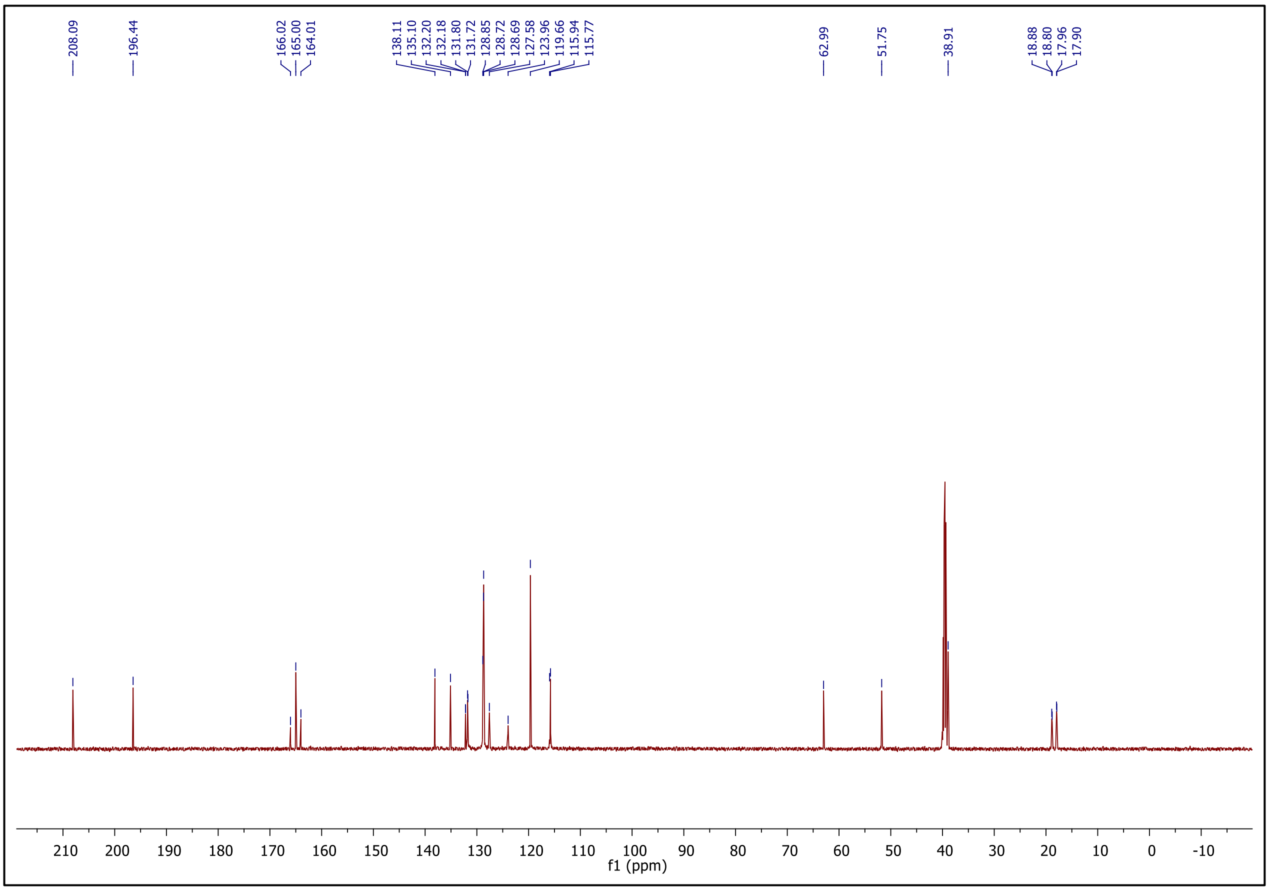
208.09
196.44
166.02
165.00
164.01
138.11
135.10
132.20
132.18
131.80
131.72
128.85
128.72
128.69
127.58
123.96
119.66
115.94
115.77
62.99
51.75
38.91
18.88
18.80
17.96
17.90
f1 (ppm)

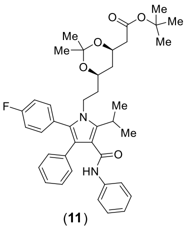
F
Me
Me
O
O
Me
Me
Me
O
O
N
Me
Me
O
HN

(**11**)

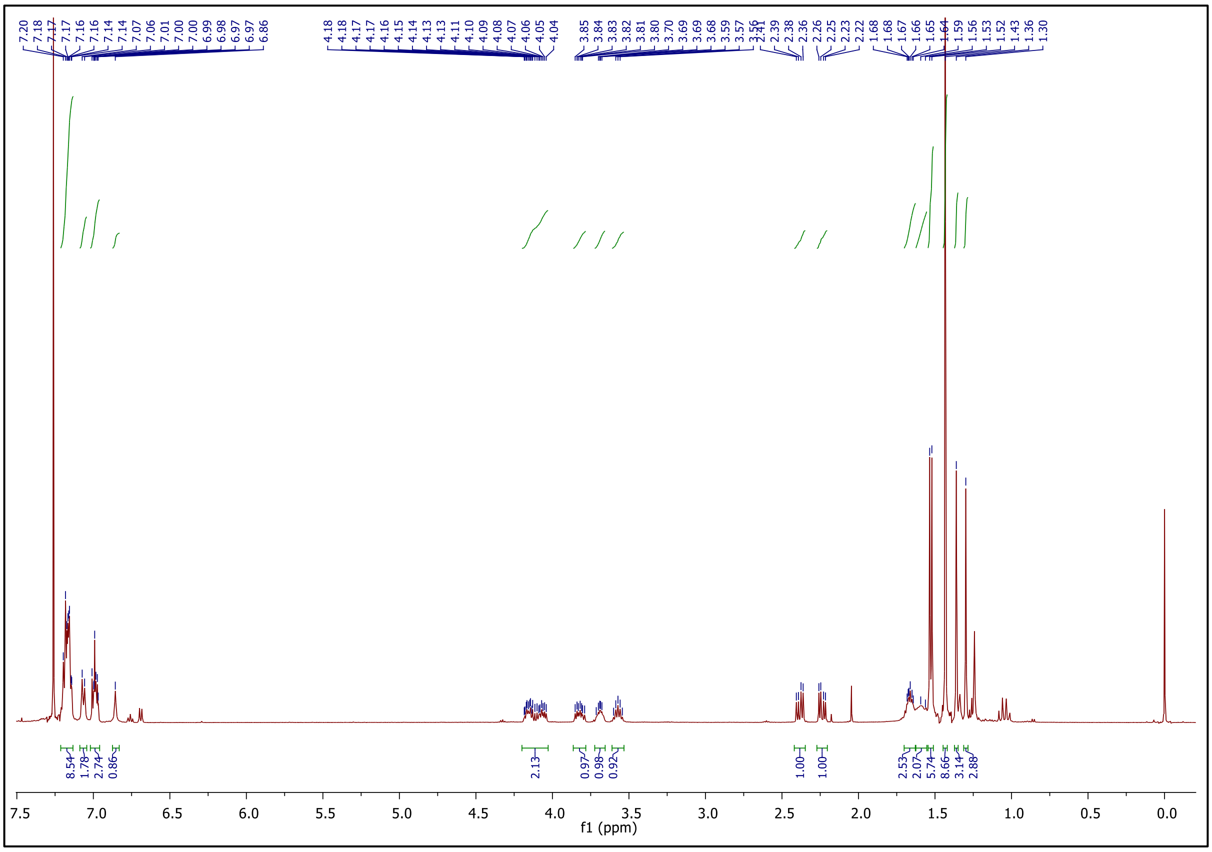
7.20 7.18 7.17 7.17 7.16 7.16 7.14 7.07 7.06 7.01 7.00 7.00 6.99 6.98 6.97 6.97 6.86
4.18 4.18 4.17 4.17 4.16 4.15 4.14 4.13 4.13 4.13 4.11 4.10 4.09 4.08 4.07 4.06 4.05 4.04
3.85 3.84 3.83 3.82 3.81 3.80 3.70 3.69 3.69 3.68 3.59 3.57 3.56
2.41 2.39 2.38 2.36 2.26 2.25 2.23 2.22
1.68 1.68 1.67 1.66 1.65 1.64 1.59 1.56 1.53 1.52 1.43 1.36 1.30
8.54 1.78 2.74 0.86
2.13
0.97 0.98 0.92
1.00 1.00
2.53 2.07 5.74 8.66 3.14 2.88
7.5 7.0 6.5 6.0 5.5 5.0 4.5 4.0 3.5 3.0 2.5 2.0 1.5 1.0 0.5 0.0
f1 (ppm)

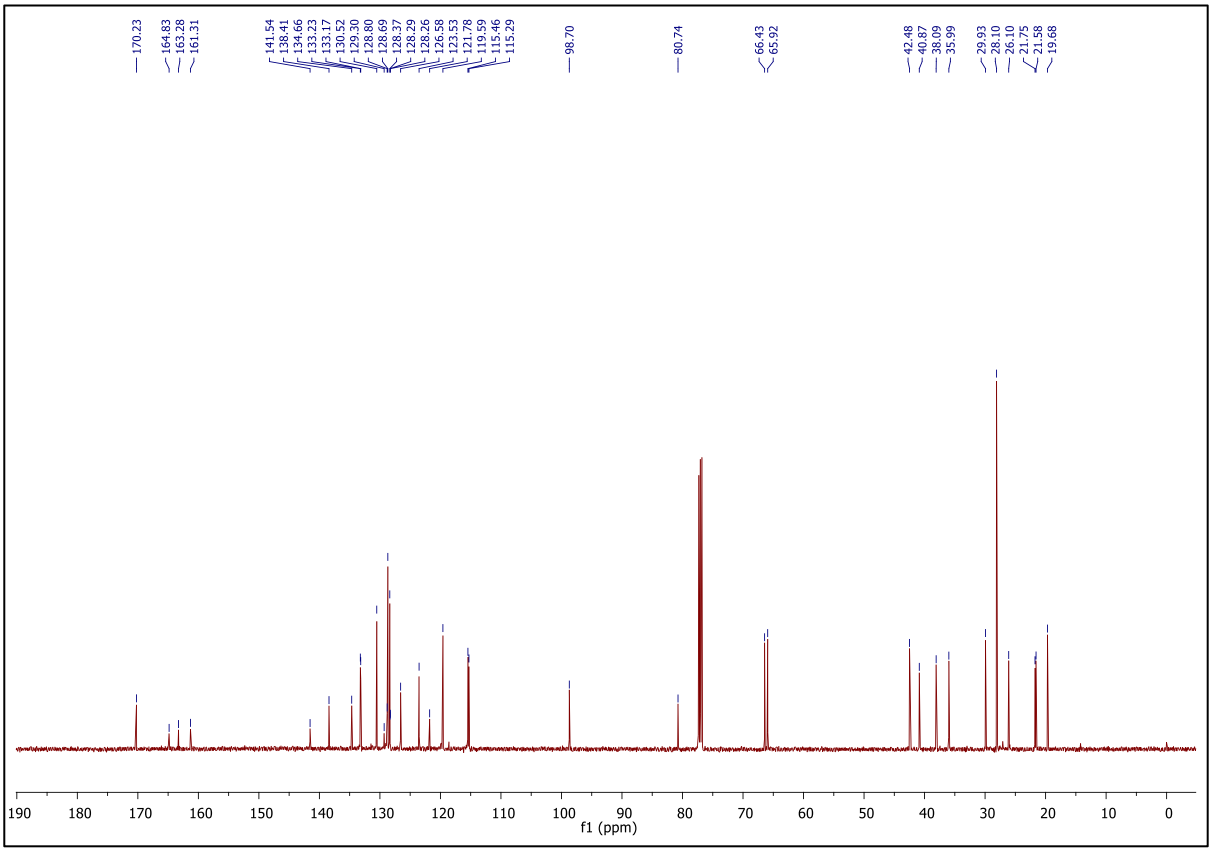
170.23
164.83 163.28 161.31
141.54 138.41 134.66 133.23 133.17 130.52 129.30 128.80 128.69 128.37 128.29 128.26 126.58 123.53 121.78 119.59 115.46 115.29
98.70
80.74
66.43 65.92
42.48 40.87 38.09 35.99
29.93 28.10 26.10 21.75 21.58 19.68
190 180 170 160 150 140 130 120 110 100 90 80 70 60 50 40 30 20 10 0
f1 (ppm)

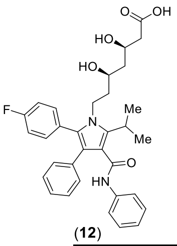

(**12**)

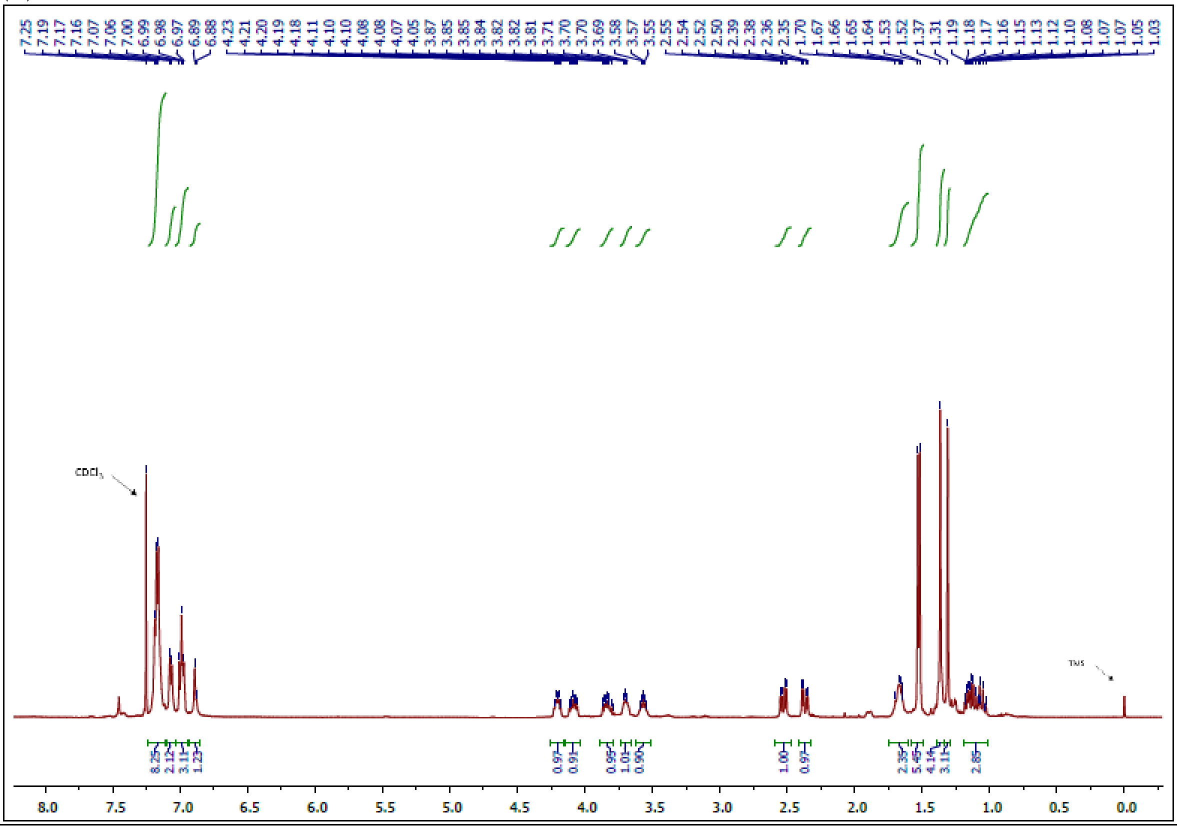

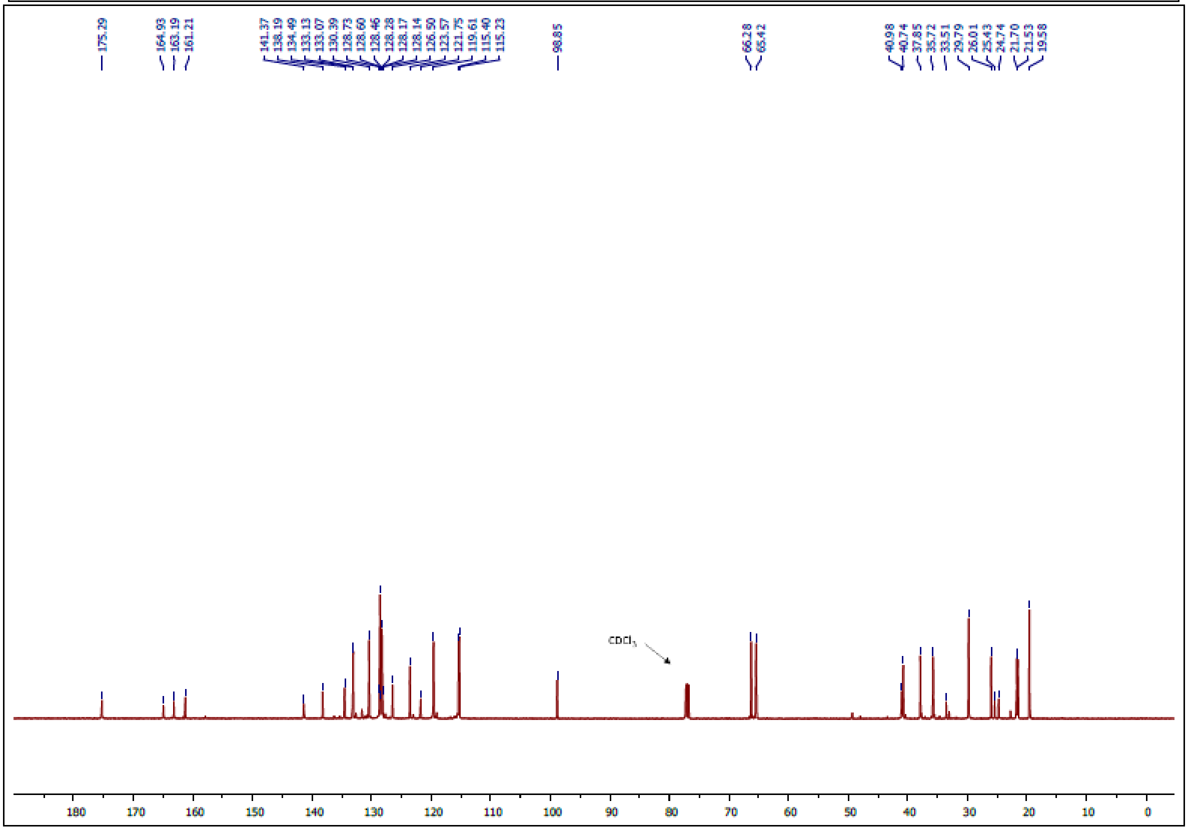

Characterization of [^{18}F]atorvastatin (radio-HPLC and radio-TLC)

Final product **[^{18}F]12** was purified and characterized by radio-HPLC (SymmetryPrep™ C18 7 µm 7.8x300 mm column; A: sodium acetate 0.05 M pH 4.7, B: acetonitrile; 0-4 min.: 90% A, 4-15 min.: 90% A to 20% A, 15-25 min.: 20% A to 5% A, 25-33 min.: 5% A 33-34 min.: 5% A to 90% A, 34-35 min.: 90% A; flow: 6 mL/min., Rt ([^{18}F]12) ≈ 16 min., Rt ([^{18}F]11) ≈ 24 min.). UV spectrophotometer for evaluation of chemical species was tuned to 244 nm in accordance with European Pharmacopoeia recommendations. Formation of **[^{18}F]12** was also assessed by radio-TLC (TLC-SG developed with ethanol:sodium phosphate 0.1 M pH 7.4 (65:35 v:v), Rf ([^{18}F]F^{-}) = 0.0-0.1, Rf ([^{18}F]11) = 0.5-0.6 and Rf ([^{18}F]12) = 0.8-0.9).

Radio-HPLC profile of [^{18}F]atorvastatin:

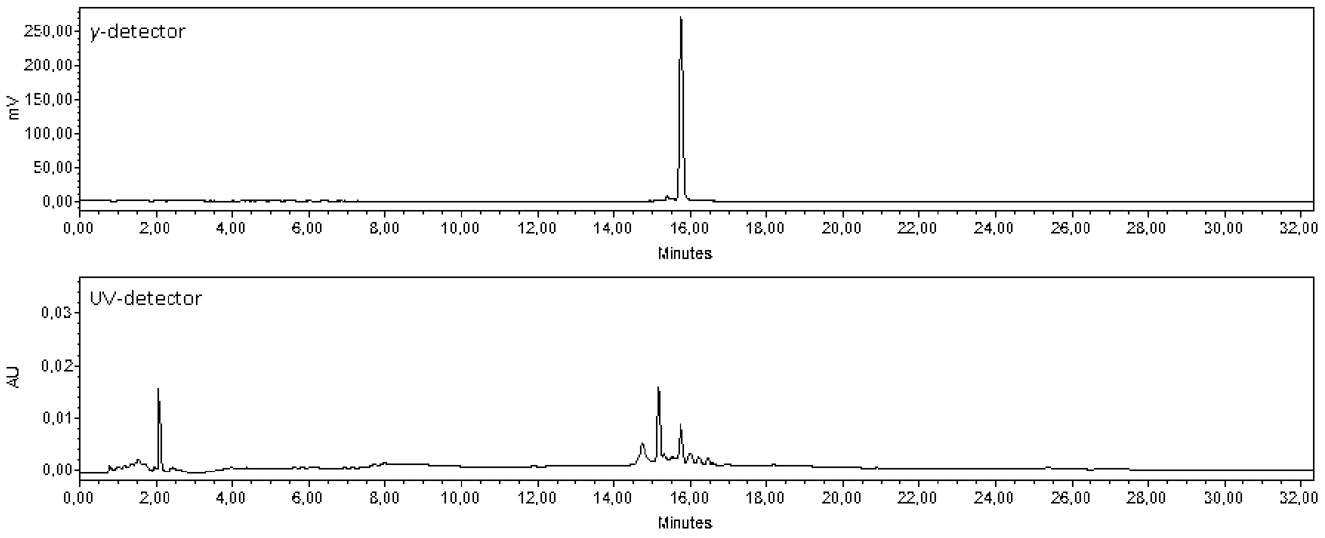

Radio-HPLC profile of [^{18}F]atorvastatin spiked with nonradioactive standard:

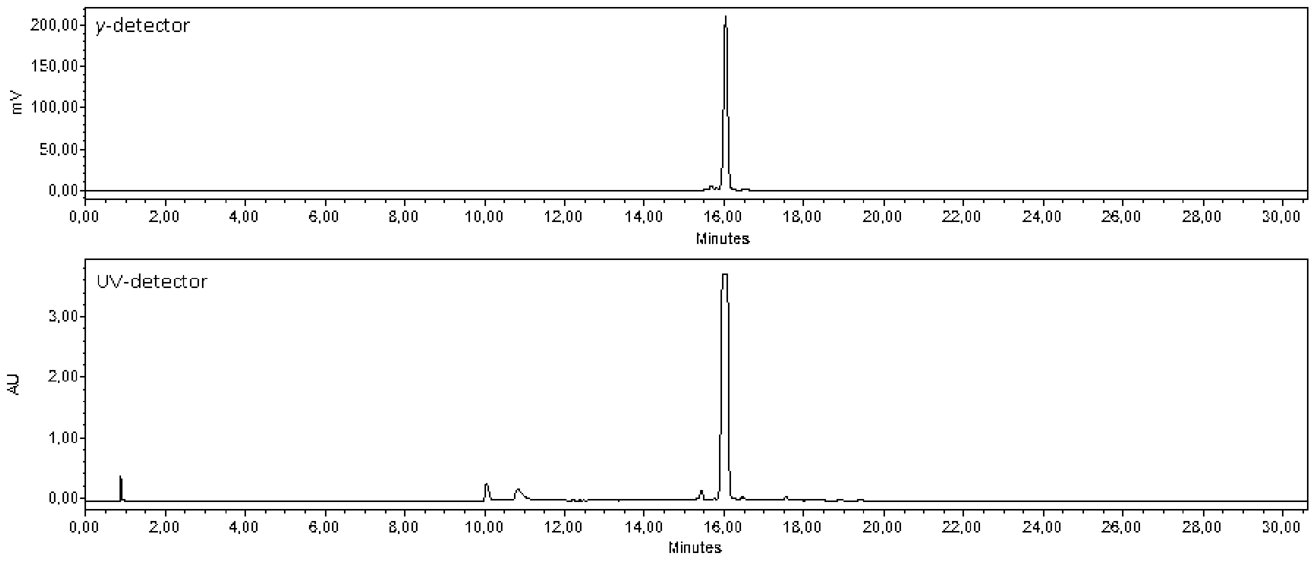

TLC and radio-TLC profile of non-radiolabelled standards and radiochemical species:

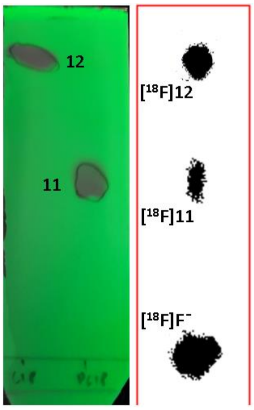

[18F]atorvastatin QC and molar activity calculation:

For the quality control (QC) of the final radiotracer and assessment of the molar activity, a radio-UPLC system was used (ACQUITY HSS T3 1.8 µm 3.0x50 mm column; A: sodium acetate 0.01 M in H_2O:MeOH:ACN 9:0.6:0.4 v:v:v, B: sodium acetate 0.01 M in H_2O:MeOH:ACN 1:5.4:3.6 v:v:v; 0-2 min.: 100% A, 2-5 min.: 100% A to 40% A, 5-6 min.: 40% to 0% A, 6-9 min.: 0% A, 9-10 min.: 0 to 100% A; flow: 0.8 mL/min.; UV 244 nm, Rt ([18F]12) ≈ 6.2 min.). A calibration curve was drawn with atorvastatin standard (6 points, averaged triplicate, r^2 = 0.9993) to estimate molar activity (GBq/µmol) of the final [18F]atorvastatin.

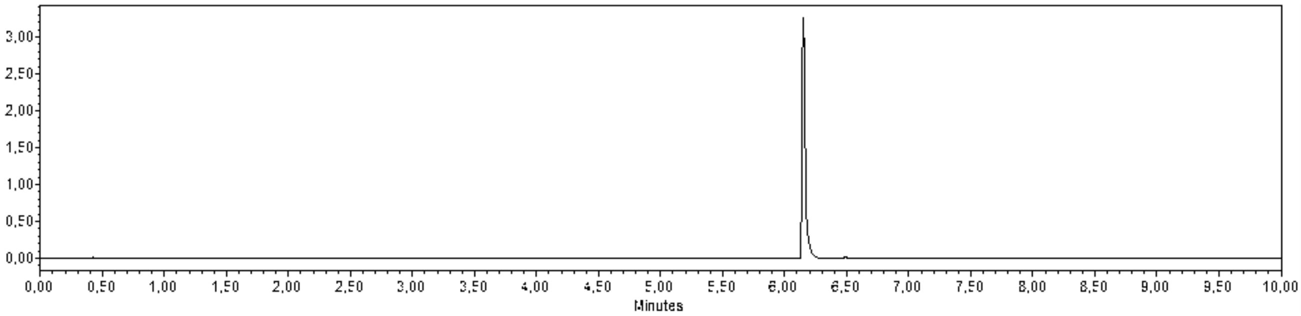

[18F]atorvastatin stability up to 4 hours in human serum assessed by radio-TLC:

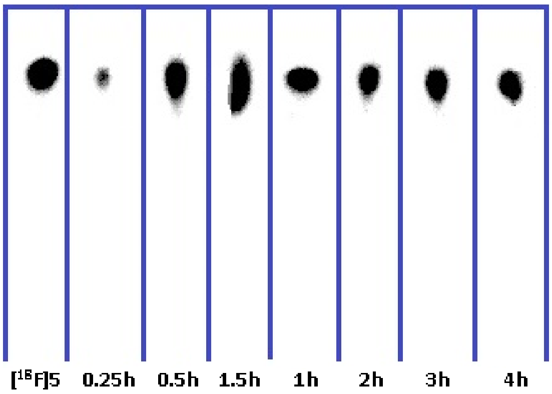

Data from autoradiographic experiments

Digital luminescence units (DLU) /mm^2 values from in vitro autoradiography with [^{18}F]atorvastatin on rat liver tissue counterparts without (control) and with (blocked) atorvastatin pre-treatment:

	Control (non-blocked) DLU /mm^2			Blocked (atorvastatin pretreatment) DLU /mm^2		
	1	2	3	1	2	3
Liver 1	20400750	23495113	18192910	8643706	8171512	6792956
Liver 2	27549623	16945217	14260013	10082364	9625596	4213490
Liver 3	41956567	22122536	22470227	19979196	7121519	7661816
Liver 4	14294732	17251878	23440603	7244306	6341607	8681208

DLU / mm^2 values from in vitro autoradiography with [^{18}F]atorvastatin on the aorta counterparts of a normal and atherosclerotic rat model without (control) and with (blocked) atorvastatin pre-treatment:

	Normal rat aorta DLU /mm^2		Atherosclerotic rat aorta DLU /mm^2	
Aorta sections	Control	Blocked	Control	Blocked
(aortic arch) upper extermity	1341489	907018	5070901	1448214
↓	2040292	808135	4811672	1798011
	2038482	1245185	2615112	989977
	2179488	1311602	2625597	777821
	2557724	863490	4191145	1696952
	1375438	728861	2753193	2006106
abdominal extermity	1985630	748847	2728067	1562202
